# Setting Ranges in Potential Biomarkers for Type 2 Diabetes Mellitus Patients Early Detection By Sex—An Approach with Machine Learning Algorithms

**DOI:** 10.3390/diagnostics14151623

**Published:** 2024-07-27

**Authors:** Jorge A. Morgan-Benita, José M. Celaya-Padilla, Huizilopoztli Luna-García, Carlos E. Galván-Tejada, Miguel Cruz, Jorge I. Galván-Tejada, Hamurabi Gamboa-Rosales, Ana G. Sánchez-Reyna, David Rondon, Klinge O. Villalba-Condori

**Affiliations:** 1 Unidad Académica de Ingeniería Eléctrica, Universidad Autónoma de Zacatecas, Jardín Juárez 147, Zacatecas 98000, Mexico; alejandro.morgan@uaz.edu.mx (J.A.M.-B.); hlugar@uaz.edu.mx (H.L.-G.); ericgalvan@uaz.edu.mx (C.E.G.-T.); gatejo@uaz.edu.mx (J.I.G.-T.); hamurabigr@uaz.edu.mx (H.G.-R.); agsreyna@uaz.edu.mx (A.G.S.-R.); 2 Unidad de Investigación Médica en Bioquímica, Hospital de Especialidades, Centro Médico Nacional Siglo XXI, Instituto Mexicano del Seguro Social, Ciudad de México 06720, Mexico; mcruzl@yahoo.com; 3 Departamento de Estudios Generales, Universidad Continental, Arequipa 04001, Peru; drondon@continental.edu.pe; 4 Vicerrectorado de Investigación, Universidad Católica de Santa María, Yanahuara 04013, Peru; kvillalba@ucsm.edu.pe

**Keywords:** machine learning, biomarkers, type 2 diabetes, Akaike information criterion, recursive feature elimination

## Abstract

Type 2 diabetes mellitus (T2DM) is one of the most common metabolic diseases in the world and poses a significant public health challenge. Early detection and management of this metabolic disorder is crucial to prevent complications and improve outcomes. This paper aims to find core differences in male and female markers to detect T2DM by their clinic and anthropometric features, seeking out ranges in potential biomarkers identified to provide useful information as a pre-diagnostic tool whie excluding glucose-related biomarkers using machine learning (ML) models. We used a dataset containing clinical and anthropometric variables from patients diagnosed with T2DM and patients without TD2M as control. We applied feature selection with three different techniques to identify relevant biomarker models: an improved recursive feature elimination (RFE) evaluating each set from all the features to one feature with the Akaike information criterion (AIC) to find optimal outputs; Least Absolute Shrinkage and Selection Operator (LASSO) with glmnet; and Genetic Algorithms (GA) with GALGO and forward selection (FS) applied to GALGO output. We then used these for comparison with the AIC to measure the performance of each technique and collect the optimal set of global features. Then, an implementation and comparison of five different ML models was carried out to identify the most accurate and interpretable one, considering the following models: logistic regression (LR), artificial neural network (ANN), support vector machine (SVM), k-nearest neighbors (KNN), and nearest centroid (Nearcent). The models were then combined in an ensemble to provide a more robust approximation. The results showed that potential biomarkers such as systolic blood pressure (SBP) and triglycerides are together significantly associated with T2DM. This approach also identified triglycerides, cholesterol, and diastolic blood pressure as biomarkers with differences between male and female actors that have not been previously reported in the literature. The most accurate ML model was selection with RFE and random forest (RF) as the estimator improved with the AIC, which achieved an accuracy of 0.8820. In conclusion, this study demonstrates the potential of ML models in identifying potential biomarkers for early detection of T2DM, excluding glucose-related biomarkers as well as differences between male and female anthropometric and clinic profiles. These findings may help to improve early detection and management of the T2DM by accounting for differences between male and female subjects in terms of anthropometric and clinic profiles, potentially reducing healthcare costs and improving personalized patient attention. Further research is needed to validate these potential biomarkers ranges in other populations and clinical settings.

## 1. Introduction

Type 2 Diabetes Mellitus (T2DM) is a common chronic disease that often occurs together with other comorbidities such as diabetic kidney disease (DKD) or heart disease, and poses a significant public health challenge. The most recent report from the International Diabetes Federation states that in 2021 the worldwide occurrence of T2DM among adults was 536.6 million individuals, accounting for 10.5% of the global population. It is projected that by 2045 there will be an estimated 783.2 million people living with diabetes, or 12.2% of the global population [1]. T2DM is associated with a higher risk of cardiovascular disease, kidney disease, blindness, and lower limb amputations [2]. In Mexico, according to the 2021 mortality data provided by the Instituto Nacional de Estadística y Geografía INEGI, 142,546 deaths by diabetes mellitus were reported, of which 72,324 correspond to deaths of men and 70,219, plus 3 non-specific, in the range from 0 to 85 years. Of this wide range, the numbers in the age range of 55 to 85 years or more were over 10,000 deaths [3]. T2DM is a chronic metabolic disorder characterized by high levels of blood glucose due to either insufficient insulin secretion by the pancreas or resistance to insulin action in the body. The prevalence of T2DM is increasing globally due to aging populations, sedentary lifestyles, and unhealthy diets [4].

Early detection and management of T2DM and its progression to a comorbidity are crucial to prevent complications and improve health patient outcomes. Early detection of T2DM can help prevent or delay complications [5]. The effectiveness of screening for T2DM can lead to earlier detection of T2DM, improved glycemic control, and reduced rates of complications [6]. Anthropometric features such as body mass index (BMI), waist-to-hip ratio (WHR), and waist-to-height ratio can be useful as prediction factors; due to the nature of each feature, one may be more useful than the others [7]. Other anthropometric features based on blood pressure and glycosylated hemoglobin (HbA1c) measurements provide several statistically significant differences that can be identified in relation to ethnicity [8]. Metabolomics is a promising approach to identifying biomarkers that could help in early detection and prevention of T2DM and DKD comorbidity. Metabolomics has been used to identify potential biomarkers for T2DM. One study developed a protocol based on pharmacometabolomics, nuclear magnetic resonance, and mass spectrometry (MS) to determine the best approach for treatment based on metabolomic biomarkers. Similar approaches have been used to predict the progression of T2DM using MS imaging, a promising technology that uses video and image processing to identify and provide precise information about molecules on tissue surfaces [9]. However, the high-dimensional and complex nature of metabolomics data and high cost of the process for obtaining such data poses a challenge in identifying relevant biomarkers. Early detection of T2DM through ML models is possible with datasets that provide feature values recollected through a specific window of time. Such datasets can also be improved with boosting techniques such as XGBoost or LightGBM to make more precise predictions if data are limited [10]. As procedures such as glycerolipid panels are complex, and ten times higher in cost than a lipid panel or complete blood count (though prices vary in each country depending on factors such insurance policies, laboratories, and healthcare institutions), the need for less invasive and low cost analysis arises.

The clinical manifestations of metabolic syndrome are underlying physiological processes that are often overlooked. This oversight is becoming increasingly significant, especially given the concerning rise in prevalence among young females. In this concise aspect, there are distinctions between males and females concerning the epidemiology, causes, biological mechanisms, and clinical presentation of the metabolic syndrome. Specifically, notable male and female differences encompass differing rates of dysglycemia, disparities in fat distribution, variances in adipocyte size and function, hormonal regulation of body weight and fat accumulation, and impact of estrogen decline on risk factors, among others [11]. These metabolic alterations can emphasize a direct relation between the metabolomic profile, sex, T2DM, and its comorbidities. Patients with metabolic syndrome present variations in the occurrence of abnormal blood sugar levels between males and females; these alterations increase the risk of suffering T2DM, the function and size of fat cells (or adipocytes), and hormonal mechanisms governing body weight and fat storage, all of which exhibit sex-related distinctions. Another study conducted a comprehensive metabolomic analysis using serum samples obtained from both normal subjects and subjects diagnosed with severe COVID-19, with a focus on distinguishing between males and females. This analysis revealed noteworthy variations in metabolic pathways between male and female COVID-19 patients which were not observed in control patients. These distinctions primarily centered around the lipid metabolism, pentose phosphate pathway, bile acid metabolism, and microbiome-related processing of aromatic amino acids such as tryptophan and tyrosine. The application of unsupervised statistical methods, uncovered significant sexual dimorphism in the relationships between specific clinical parameters of the patients and their overall metabolic profiles [12].

According to Allen A. et al., DKD tends to manifest in about half of T2DM patients. The focus on diabetes research must be to aim to overcome the challenges associated with delayed diagnosis, which often result from inconsistent screening practices. This study implemented decision trees (DT) and two variations of this model, random forest (RF) and XGBoost, capable of predicting different stages of DKD within a 5-year timeframe following the initial diagnosis of T2DM. The results indicated that the models surpassed the performance of the Centers for Disease Control and Prevention risk score in both the hold-out test and external datasets [13]. Another investigation conducted by Chan L. et al. enhanced the prediction of progression in DKD by formulating and validating a machine-learned prognostic risk score named KidneyIntelX™. This innovative model integrates electronic health records and biomarkers through the training of an RF model. The model was evaluated based on the area under the curve (AUC), positive and negative predictive values, and net reclassification index. A comparative analysis was executed against a conventional clinical model and the DKD: Improving Global Outcomes categories. This research aimed to predict a composite outcome, specifically, a decline in the estimated glomerular filtration rate (GFR) of ≥5 mL/min per year, ≥40% sustained decline, or kidney failure within a span of 5 years. The correlation between the derivation and validation of the ML model was instrumental in gauging its efficacy and reliability in predicting crucial outcomes in DKD progression [14].

Belur-Nagaraj S. et al. investigated the potential of using the kidney age index (KAI), a measure of the deviation between biological age (BA) and chronological age (CA), to assess kidney function in patients with DKD. The KAI was developed by training ML algorithms such as logistic regression (LR), RF, SVM, and feed-forward neural network (FNN) on three datasets (PREVEND, RENAAL and IDNT) of healthy individuals and DKD patients. The FNN algorithm had the best performance in predicting CA based on various clinical markers, which were then used to predict BA for each patient. The KAI was calculated as the difference between BA and CA. The researchers found that the KAI was significantly higher in patients with DKD than in healthy individuals. This suggests that DKD accelerates the aging process, leading to a higher BA than would be expected based on CA [15]. Osteopontin (OPN), a biomarker presented by Moszczuk B. et al., has emerged as a promising candidate for DKD detection. The research included patients with various biopsy-proven glomerulopathies (GNs) such as immunoglobulin A DKD (IgAN), membranous DKD, and lupus nephritis, as well as control group. Utilizing Boruta and RF revealed that OPN exhibited a significant increase in IgAN compared to other GNs at baseline, enabling the development of a ML algorithm with 87% accuracy in differentiating IgAN from other GNs based solely on urinary OPN levels [16]. The prediction of DKD in a study conducted by Shuo-Ming O. et al. included LR, extra tree classifier, RF, gradient boosting DT, XGBoost, and LightGBM to predict the risk of developing end-stage renal diseases in newly diagnosed T2DM. The results pointed out in this research shorten the gap between each disease prediction and straighten the early diagnosis, preventing more severe comorbidities [17].

Rodriguez-Romero V. et al. conducted a retrospective analysis of the Action to Control Cardiovascular Risk in Diabetes trial. The research focused on differentiating early and late predictors by stratifying longitudinal data based on time after patient enrollment. Among the evaluated algorithms, the RF and simple LR methods demonstrated superior performance, as key baseline factors such as GFR, urinary creatinine, urinary albumin, potassium, cholesterol, low-density lipoprotein (LDL), and urinary albumin-to-creatinine ratio were identified as DKD predictors. Early predictors included baseline values of GFR, systolic blood pressure (SBP), fasting plasma glucose (FPG), and potassium at month 4 [18]. Another retrospective cohort study conducted by Cheng-Chieh L. et al. aimed to develop a DKD and heart failure prediction model for T2DM patients. Following Framingham Heart Study procedures, a Cox proportional hazard regression model identified key predictors. The risk scoring incorporated factors such as age, sex, diabetes onset, blood pressure, medications, creatinine, HbA1c, SBP, retinopathy, albuminuria, diabetes medications, and hyperlipidemia. This model demonstrated high accuracy and discrimination for prediction, offering valuable screening potential for early prevention in T2DM patients [19].

The characteristics identified as risk factors for diabetic DKD encompass various elements. These comprise urinary albumin excretion, glucose levels, blood pressure, dyslipidemia, obesity, smoking, duration of diabetes, age, sex, and retinopathy. Additionally, recognized risk factors involve oxidative stress, inflammation, genetic background, ethnicity, and glomerular hyperfiltration [20]. These diverse factors collectively contribute to the intricate landscape of DKD susceptibility. Understanding and addressing these multifaceted determinants is crucial for comprehensive management and prevention strategies. A nomogram presented in a Chinese study contributed to this assessment by presenting a list of features as predictors, including SBP, diastolic blood pressure (DBP), FPG, HbA1c, triglycerides, serum creatinine, blood urea nitrogen, and BMI [21]. All of these features have been previously presented as biomarkers not just for the DKD but as biomarkers to predict T2DM in the early stages, creating a focus for diverse studies to set control of these features as treatment for these diseases.

The features presented in each paper can vary due to ethnicity and the balance of the dataset. Diverse studies have used HbA1c as a key measure of average blood glucose over the past 2–3 months due to its common use in diabetes management. FPG is another example, consisting of a test that measures glucose levels after an overnight fast to provide insights into fasting glucose concentrations. Creatine Kinase-MB (CK-MB) is an enzyme found in the heart; CK-MB levels help to diagnose heart attacks and other heart-related conditions. Diverse proteins associated with high density lipoprotein (HDL) cholesterol play different roles in lipid metabolism. Finally, total cholesterol, a standard blood test measuring the total amount of cholesterol in the blood, includes both HDL and LDL cholesterol [22].

Lipids have a role in the interplay between viral infections, host metabolic responses, and immune reactions. Focusing on COVID-19, an investigation by Castañé H. et al. contrasted the lipidomic signatures of COVID-19-positive patients with those of COVID-19-negative patients with inflammatory diseases, using healthy volunteers as control. They leveraged liquid chromatography coupled with mass spectrometry and ML tools for nuanced data interpretation, unraveling distinct lipidomic profiles associated with COVID-19 and other inflammatory conditions [23]. In the context of Mexico, ranking fifth globally in COVID-19 deaths, a study by Rojas-García M. leveraged ML to identify risk markers for lethality. XGBoost emerged as the most effective model, demonstrating high sensitivity and specificity. For females, diabetes and arthralgia were key markers, while DKD and chest pain were crucial for males. Dyspnea, hypertension, and polypnea were universal risk factors; lastly, age proved the most influential demographic factor for lethality. These markers offer valuable insights for initial triage, especially in resource-constrained regions [24].

In other ML implementations, Agliata A. et al. presented an innovative approach utilizing an ANN for predicting T2DM, offering significant promise for disease management and prevention. This study employed a binary classifier trained from scratch, exploring previously unknown associations between health parameters and diabetes onset. They leveraged three datasets: the NHANES biennial survey, MIMIC-III, and MIMIC-IV. The research showcased the superior long-term predictive capability of ANNs. This work established a foundation for accurate risk assessment and early detection, contributing to advancements in diabetes prevention strategies [25]. Dichotomic studies have also shown promising results and provided a different set of tools for diagnosis, prognosis, and early detection of diseases. LASSO with Glmnet provides a versatile regression model for predicting undiagnosed T2DM. In a study that evaluated ML-based models including RF, XGBoost, and LightGBM against traditional regression models utilizing 6-month batches of simulated incoming data, Glmnet showed improvement with additional data, while LightGBM demonstrated the highest variable selection stability over time, showcasing its robust predictive capabilities in T2DM diagnosis [10]. A study by Frimpong E. A. introduced a feed-forward artificial neural network (FFANN) model tailored for numeric and textual datasets to address the limitations of existing ANN models in medical diagnostics. With an optimized architecture, this FFANN maximized the number of layers and nodes for effective dataset feature learning while mitigating underfitting and overfitting issues. The proposed model exhibited improved accuracy, providing a valuable tool for disease detection in patients with diabetes within the broader context of molecular medicine [26].

Novel approaches using feature selection in diabetes prediction utilizing the LASSO method have been developed as well. Leveraging a dataset with variables such as age, sex, BMI, blood sugar levels and lifestyle factors can help to demonstrate the efficiency of LASSO in selecting significant features for diabetes prediction. The identified features can be instrumental in constructing accurate ML models for diabetes prognosis, contributing to more effective diagnosis and management strategies [27]. The application of the LASSO sparse learning model has proven effective in uncovering epidemiological patterns associated with diabetic retinopathy (DR) and other comorbidities. Studies have utilized ML to predict DR risk based on health records, highlighting LASSO as an excellent choice for high-dimensional electronic health record analysis and demonstrating its efficacy in terms of both discriminative power and variable selection while emphasizing its potential in advancing understanding and prediction of DR within healthcare analytics [28]. Leveraging the LASSO method to identify crucial risk factors can lead to comprehensive comparisons with other algorithms such as LR, RF, SVM, etc., after which multivariate LR analysis can be employed to construct nomograms for individualized prediction. With evaluation through AUC and calibration, LASSO’s superiority has been proven for diabetes risk prediction. Notably, the nomograms include specific factors for prediabetes and its progression to diabetes, demonstrating robust discrimination confirmed by well-fitted calibration curves [29].

A study by Singh Y. and Tiwari M. achieved a remarkable 93% classification accuracy on the Pima Indian Women dataset. The authors proposed a hybrid framework that mitigates issues with ill-posedness, overfitting, and class imbalance problems and employing data mining techniques, specifically, a combination of the LASSO regression algorithm for variable selection and regularization with an ANN classifier. By effectively reducing computing time through LASSO regression, this hybrid approach offers enhanced predictability and interoperability. The findings suggest that such data-mining methodologies hold substantial promise in aiding clinicians in making accurate diabetes diagnoses, contributing to improved medical decision-making [30].

Other studies have used GAs as feature selection tools. Recent studies have combined research focuses on optimizing diabetes detection models by integrating two feature selection techniques, such as the AIC and GA, which can be paired with prominent classifier algorithms to demonstrate superior performance [31]. Addressing the crucial task of selecting differential metabolites is vital for both biological and clinical applications; nevertheless, there are thousands of such metabolites, and a feature selection approach is needed to develop complex datasets for metabolomic analysis, leveraging the effectiveness of models such as SVM as a basic classifier. A method designed by Lin X. et al. employs SVM RFE for meaningful feature identification, while GA and RF are utilized to capture metabolite interactions and individual performance, respectively. When validated on a plasma metabolomics dataset of rat liver diseases, the approach identified 31 important metabolites, showcasing a synergistic effect of the three selection methods for comprehensive metabolic insights across different liver diseases [32]. This technique has been applied to human datasets as well. The GA can identify an optimal set for detecting T2DM, while utilizing a model such as RF-based GA (miRDM-rfGA) as a feature selection algorithm can generate subsets and compare them with settings using traditional feature selection methods (F-test and LASSO) [33].

In the ML community, there is a growing trend towards employing complex models, particularly in fields such as computer vision and natural language processing (NLP). These complex models often demand substantial computational resources. However, simpler models can still be highly effective, especially when data are abundant. In this case, feature selection becomes crucial to enhancing model accuracy. The RFE with cross-validation approach for predicting T2DM aims to boost classification accuracy. To overcome challenges such as overfitting, this technique incorporates additional preprocessing methods. The results can then be evaluated with classical ML algorithms such as LR, ANN, Naïve Bayes, SVM, and DT [34]. A study by Sabitha E. and Durgadevi M. aimed to underscore the significance of data preprocessing, feature selection, and data augmentation in disease prediction, using techniques in these domains to enhance the efficacy of classification algorithms in diabetes diagnosis and prediction by employing a proposed method on the Pima Indian dataset. A systematic three-category comparison revealed that data preprocessing, RFE with RF regression feature selection, and SMOTE oversampling augmentation achieved notable accuracy scores over 80% among six classifiers (LR, RF, DT, SVM, Naïve Bayes, and KNN) [35]. RFE with RF was employed for significant feature selection, with estimation revealing a robust association of diabetes with BMI and glucose level extracted using the a priori approach. These approaches can be compared to the ANN approach, offering potential support to medical professionals in treatment decisions [36].

RFE has been compared with other feature selectors using the Boruta package and a GA applied to the well-known Pima Indian diabetes dataset. It was found that Boruta outperforms them using the DT ID3 algorithm. These results, presented by Sadhasivam J. et al., indicate that applying feature selection algorithms on a standard dataset such as the Pima Indian diabetes dataset yields no change in accuracy, suggesting that the information in the standard dataset is already preprocessed and does not require further processing. However, when applied to a dataset collected from a local hospital, these feature selection methods significantly increased model accuracy [37]. Based on accuracy, the Boruta package is the most effective feature selection algorithm for the local dataset of diabetes infection; nevertheless, these results can be refuted, as the only model used for validation was DT.

ML models have shown promising results in detecting and predicting the onset of chronic diseases. The use of ML models in healthcare has become increasingly popular due to their ability to analyze large amounts of data and provide accurate predictions. The following are the insights that led this research:

- Early Detection: ML models can analyze various parameters, such as biomarkers, clinical data, and medical history, to predict the onset of conditions in patients. By identifying patients at high risk, healthcare providers can take proactive measures to prevent the development of diseases or intervene early to manage conditions.
- Personalized Medicine: ML models can help in the development of personalized treatment plans for patients. By analyzing data such as genetic information, lifestyle, and medical history, ML models can identify the most effective treatment plan for individual patients, reducing the risk of adverse reactions and improving treatment outcomes.
- Predictive Analytics: ML models can be used to predict the likelihood of complications associated with various conditions. By analyzing patient data and identifying the factors that contribute to the development of complications, healthcare providers can take proactive measures for prevention or management, reducing the burden on the healthcare system.
- Remote Monitoring: ML models can be used to remotely monitor patients, reducing the need for frequent hospital visits. By analyzing data such as vital signs and biomarkers, ML models can alert healthcare providers to potential health issues and prompt them to take necessary action.

Feature selection is a pivotal step in building effective models, particularly in the realm of medical decision-making. When dealing with medical databases, a prudent analysis and preprocessing stage is essential, as using these datasets directly can often yield counterproductive results. In this context, feature selection is the method by which the most pertinent attributes are meticulously chosen from the database. This process is instrumental in constructing robust learning models and subsequently enhancing the performance of models used in decision-making within the medical domain. In the field of biomedical research, the primary objective of feature selection is to pinpoint clinically significant and statistically robust variables while excluding unrelated or noisy ones. Numerous methods are available for feature selection, each with its own set of advantages and limitations. For instance, the stepwise approach iteratively adds the best variable in each cycle, typically yielding an acceptable set of variables. However, it can be constrained by a tendency to become stuck in local optima. Conversely, the best subset approach systematically explores the entire covariate pattern space, but can become unwieldy when dealing with datasets containing tens to hundreds of variables, which is a common scenario in today’s clinical data environment. GAs offer a solution as heuristic optimization methods for variable selection in multivariable regression models. This tutorial paper serves the crucial purpose of presenting a step-by-step guide to utilizing a GA for feature selection. It provides not only theoretical insights but also practical implementation in the form of R code that can be adapted to various data analysis requirements. The implementation of feature selection using GA, specifically with GALGO, is of paramount importance. GALGO offers a systematic and efficient approach to choosing the most relevant features from complex medical datasets. This method, as demonstrated in the present paper, is instrumental in improving the performance of predictive models, ensuring that only the most clinically significant variables are included and that irrelevant noise variables are excluded. This has a direct impact on the quality and reliability of medical decision-making processes, making for a crucial contribution to the field of healthcare and medical research [38].

FS offers an efficient approach for reducing the dimensionality of high-dimensional data. The rationale for employing sequential forward selection lies in its effectiveness in revealing the intricate interactions between features. Sequential forward selection is a wrapper feature selection method. The algorithm is initiated with an empty features set, and begins by adding the feature with the highest feature importance score to this empty set These features are then ranked based on their feature importance, allowing for extraction of the top-performing features using an extra tree classifier. Selim Buyrukoglu and Ayhan Akbasis proposed implementing the wrapper feature selection techniques and an ensemble ML model to identify biomarkers for early detection of T2DM, achieving promising results [39].

The results of this study have the potential to contribute to early detection and prevention of T2DM and its comorbidities by identifying differences between male and female patients and identifying feature combinations that could help in the development of more personalized and effective preventive and therapeutic strategies. The objective of this study is to exhibit differences between men and women in biomarkers for T2DM due to variations in physiology, hormones, and fat distribution. Men tend to accumulate visceral fat, which is linked to insulin resistance (IR), while women often have more subcutaneous fat. Testosterone influences fat distribution and insulin sensitivity in males, with lower levels increasing T2DM risk. In pre-menopausal women, estrogen is protective; however, its decline post-menopause raises risk. Men typically have higher triglycerides and lower HDL cholesterol, whereas post-menopausal women may see lipid profile deterioration. Men show higher inflammatory markers affecting T2DM risk, such as CRP, while women’s inflammatory responses vary with hormone levels. Men generally have higher blood pressure earlier in life, which is a T2DM risk factor, whereas women’s cardiovascular risk spikes post-menopause. Men often exhibit higher fasting glucose and IR, whereas women’s glycemic control fluctuates with hormonal changes [40].

This work is divided into four sections, the first of which is this introduction. Section 2 describes the data, models, and methodology used to develop and validate the ensemble model. Section 3 shows the obtained results and includes a detailed analysis using the output graphs. Finally, Section 4 provides a discussion, conclusions, and prospects for future work.

## 2. Materials and Methods

**Figure 1:**
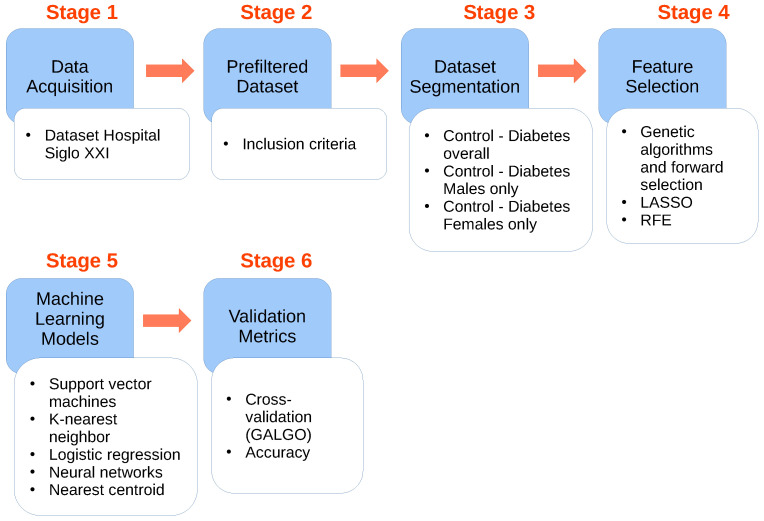
Methodology.

The methodology for this study comprises six stages, visually represented in Figure 1, and is elucidated as follows. In the initial stage, the utilization of the IMSS Siglo XXI dataset is expounded upon (depicted as Figure 1, Stage 1). Moving on to the second stage, we delve into a preliminary data analysis involving the selection of subjects based on specific inclusion criteria, denoted by Figure 1, Stage 2. Subsequently, the data within the dataset are categorized into three distinct groups, namely, Control—Diabetes overall, Control—Diabetes Males, and Control—Diabetes Females, as illustrated by Figure 1, Stage 3. The fourth stage involves the implementation of feature selection through RFE, LASSO, and GA with GALGO and FS, as detailed by Figure 1, Stage 4. The fifth stage explains the development of the ML models, including SVM, KNN and Nearcent, LR, and ANN, utilizing the primary features from the previous stages (see Figure 1, Stage 5). Finally, in order to assess the performance of our models, the models’ validation is elaborated upon, taking into account various metrics such as accuracy, sensitivity, specificity, F1, score and AUC, as represented in Figure 1, Stage 6.

The flowchart illustrating our proposed methodology is presented as follows. The blue squares represent the data analysis process, while the white squares outline the specific tasks within each step. Stage 1: To commence, the anthropometric and clinic dataset from the Hospital Siglo XXI is acquired. Stage 2: The dataset undergoes analysis, resulting in the creation of new datasets, a set of features were eliminated presented in Table 1 achieved by the selection of subjects based on the criteria outlined in Table 2 and final features included in the study shown in Table 3. Stage 3: Subsequently, the dataset observations are segmented into three distinct groups, namely, Control—Diabetes overall, Control—Diabetes Males, and Control—Diabetes Females. Stage 4: GA and FS are combined to extract essential data features, then LASSO and RFE are compared with the AIC implementation to provide the best possible model outcome to determine the model with the highest performance. Stage 5: To leverage the key features for detection of diabetes and their differences between male and female patients, multiple models were generated implementing the SVM, KNN, LR, ANN, and Nearcent techniques included in the ensemble model. Stage 6: The validation process involved the use of various metrics, including cross-validation via GALGO and average accuracy and 10-fold cross-validation for all models implemented, with a separation of 70% for training and 30% for tests. The complete flowchart is presented in Figure 1.

### 2.1. Sample

The dataset originated from the Centro Médico Nacional Siglo XXI in Mexico City. All Mexican participants provided their consent by signing an informed consent letter. The study protocol adhered to the Helsinki criteria and received approval from the Ethics Committee of Instituto Mexicano del Seguro Social under the number R-2011-785-018. The dataset includes a total of 1726 individuals, with 887 cases of T2DM and 839 control subjects. In terms of sex distribution, the dataset encompasses 855 men and 871 women. The dataset contains 41 features, including anthropometric, clinical, and identification data.

### 2.2. Data Treatment

In this phase of the process, data underwent treatment, commencing with data imputation to address missing values and features beyond the study’s scope. Subsequently, the imputed values were incorporated into the database. The final step involved normalizing the data.

### 2.3. Data Imputation

This study implemented an exclusion criteria, exclusively working with observations featuring complete data for all variables and discarding others with missing or irrelevant features. Features that could identify patients, such as IDs, were excluded, as they only represented internal patient identification data and consecutive numbers. Null or not available data (NA) were removed, particularly for features associated exclusively with T2DM-positive cases, exhibiting non-existent data in control patients, or cases where data were not recorded.

Accordingly, all medications and their daily intake amounts (Glibenclamide, Glibenclamide dose, Metformin, Metformin dose, Pioglitazone, Pioglitazone dose, Rosiglitazone, Rosiglitazone dose, Acarbose, Acarbose dose, Insulin, and Insulin dose) were eliminated. Additionally, data from three patients with missing values for the ‘hypertension under treatment’ feature were excluded. Features such as GFR, age of diagnosis (specifically for T2DM-positive cases), HbA1c, and T2DM complications were also removed given that these data were exclusively present for T2DM-positive patients.

Patients that had negative values in WHR, BMI, Creatinine, and HDLc were eliminated to preserve the integrity of the values.

The T2DM complications feature, which contains comorbidities associated with T2DM, was excluded due to being outside the experiment’s scope. It contained NA values in some cases, and was entirely NA for all control patients.

The glucose feature, a well-known biomarker, was also removed from the experiment’s scope. The reason for its exclusion was to better evaluate the performance of other features, considering its high AUC of over 90% in univariate models tests already signifies its prominence in the analyzed dataset.

The salary and education features were also removed from the scope of this study.

### 2.4. Feature Selection

The dataset employed in this research comprised a diverse array of 1726 distinct patients, and was divided again into male and female datasets, providing different features and creating subsets potentially serving as biomarkers or aiding in addressing classification challenges. However, managing this task proved computationally demanding and intricate. To tackle this complexity, we employed a GA by utilizing the GALGO R 1.4 package [41]. GALGO facilitatef feature selection for categorizing individuals as either T2DM or DN positive cases.

#### 2.4.1. Genetic Algorithm—GALGO

In our methodology, GALGO was used to initialize a population of chromosomes, each containing randomly selected sets of features; chromosomes’ fitness was assessed based on their effectiveness in accurately categorizing individuals into their respective T2DM or DN stages depending on their fitness scores, crossover, mutation, or contribution to the generation of the next offspring. This iterative process continued until specific conditions were met, such as achieving the predefined objective (set to 1 in this study) or reaching the predetermined iteration limit, in this case two times the number of observations (rounded to 2000) for all GALGO implementations of the full dataset (1726 patients) and when splitting the full dataset by male and female.

Following GALGO’s execution, the output underwent a FS procedure to identify the best-performing model or feature set This selected model or feature set was then prepared for integration into an ML model. FS, a technique often employed in GAs, was employed to enhance GALGO’s output in order to present the most optimal model.

GALGO provided flexibility concerning model criteria or parameters. In this study, we configured it to accommodate KNN, Nearcent, ANN, LR, and SVM, as specified in the relevant table in the Full Siglo XXI dataset (using 1726 patients in total) (Table 4).

With the same models for validation and using the same criteria, this study also used GALGO configured it to accommodate KNN, Nearcent, ANN, LR, and SVM, as specified in the respective tables in the Male (855 male patients) and Female (871 female) Siglo XXI datasets (Table 5).

#### 2.4.2. Forward Selection—GALGO

Another technique implemented after the GALGO runs was the FS technique. FS is a feature selection approach widely used in ML and statistical modeling. It operates by iteratively adding one feature at a time, commencing with an empty feature set. The primary aim is to identify the most pertinent and informative features that enhance the model’s performance while mitigating complexity and the risk of overfitting.

The process typically commences with initializing an empty feature set or a minimal set of features considered relevant based on prior knowledge or domain expertise. Subsequently, a feature evaluation is performed involving training a model using the selected features and gauging its performance using chosen evaluation metrics such as accuracy, F1-score, AUC, or others on a validation or cross-validation dataset.

The feature selection process then contemplates the addition of one feature from the remaining pool of features to the current set. The selection can be based on various criteria, including correlation with the target variable, *p*-values from statistical tests, or other relevant factors. The model is then reevaluated, with a new model trained using the expanded set of features and its performance assessed on the same validation or cross-validation dataset.

In order to determine whether to keep or exclude the added feature, a comparison is made between the performance of the new model with the added feature and the previous model without the feature according to the chosen evaluation metric. If the performance shows improvement, then the feature is retained in the set; otherwise, it is removed. This process is repeated iteratively through steps 3 to 5 until a predefined stopping criterion is met. This stopping criterion can be based on factors such as a predetermined number of features to be selected or a specific level of desired model performance.

When the stopping criterion is satisfied, the final set of selected features is employed in building the model [31].

#### 2.4.3. LASSO

LASSO is a feature selection technique widely used in ML for the detection of T2DM. It is a method that applies regularization to a predictive model by adding a penalty term to the loss function. This penalty encourages the model to shrink or eliminate the coefficients associated with certain features, thereby promoting feature selection and preventing overfitting.

LASSO aims to solve an optimization problem by minimizing a loss function that quantifies the error between the predicted outcomes and the actual outcomes in a dataset. The objective function can be defined as follows:

(1) $$L\left(\beta \right)=\frac{1}{2N}\sum_{i=1}^{N}{\left(y_{i}-X_{i}\beta \right)}^{2}+\lambda \sum_{j=1}^{p}\left|\beta_{j}\right|$$

where *N* represents the number of samples, $y_{i}$ is the target variable (T2DM status) for the *i*-th sample, $X_{i}$ represents the feature vector for the *i*-th sample, which includes *p* features, β is a vector of coefficients associated with each feature, and λ is the regularization parameter, which controls the strength of the penalty.

The key feature of LASSO is the addition of the L1 regularization term $\lambda \sum_{j=1}^{p}\left|\beta_{j}\right|$ to the loss function. This term encourages the coefficients (β) to be sparse, meaning that it forces many coefficients to be exactly zero. As a result, LASSO promotes feature selection by effectively eliminating irrelevant or less important features. The optimization problem is solved to find the values of the coefficients (β) that minimize the objective function. This process involves adjusting the values of β to minimize the error term while simultaneously applying the penalty term. As a result of L1 regularization, some coefficients are reduced to zero during optimization, effectively selecting a subset of the most relevant features. These selected features are considered as the most influential for the detection of T2DM [29].

#### 2.4.4. Akaike Information Criterion

The AIC is a valuable tool used for assessing the relative quality of statistical models. In addition to its application in statistical modeling, the AIC has proven to be highly effective in feature selection processes and ML applications, yielding favorable results.

In the context of feature selection, the AIC is employed to generate models by initially considering all available features in the dataset. It employs a fitting prediction technique called stepwise regression, which allows for the selective addition or removal of features from the complete feature set [42].

The use of the AIC over the AICc is because it has proven to be a better tool in models with more observations than features. It is represented as follows:

(2) $$\mathrm{AIC}=2k-2\ln(\hat{L}).$$

Information criteria are essential tools in model selection, providing a means to evaluate and compare models based on their goodness of fit and complexity. A variety of information criteria tools exist, including the Bayesian Information Criterion (BIC) and Schwarz Information Criterion, both of which incorporate a stronger penalty for model complexity compared to the AIC but cannot manage complex collections of models in high-dimensional variable selection problems. The BIC is derived from a Bayesian framework, and tends to favor simpler models compared to the AIC. Another alternative is the Deviance Information Criterion (DIC), which is used in Bayesian model comparison, especially for hierarchical models, this criterion balances goodness of fit with model complexity, similar to AIC and BIC. Another, called the Corrected Information Criterion (CIC), aims to improve on the limitations of other criteria, particularly in terms of bias correction. The CIC provides a more nuanced adjustment for small sample sizes compared to the AIC and AICc. Another comparison made in this study is with the Focused Information Criterion (FIC), which is used to select models based on their performance concerning a specific focus parameter or functional of the model. The FIC evaluates models based on a combination of bias and variance concerning the parameter of interest, providing a criterion tailored to specific objectives. Each criterion has its own strengths and is suitable for different scenarios. The AIC and AICc are generally used for frequentist approaches, while the BIC and DIC are more common in Bayesian contexts. The CIC offers enhanced small-sample adjustments, while the FIC provides a targeted model selection approach [43,44,45,46].

### 2.5. Model Development

With the features selected, the development of the models with data partitioning can begin. This process seeks to ensure that the results of the training and tests are as accurate as possible while avoiding overfitting or mismatching. The models are paired with those used in the GALGO implementations. In this way, the selection is consistent with the results of the ML implementations, as presented in the five models we implemented (Table 4).

### 2.6. Logistic Regression

The fundamental concept of LR is to model the probability of a given input *x* belonging to a particular class. The LR model estimates the probability P(y=1|x) as a function of the independent variables $x=(x_{1},x_{2},\ldots ,x_{p})$ [47].

The logistic function, also known as the sigmoid function, is used to map the predicted values to probabilities. It is defined as follows:

(3) $$\sigma \left(z\right)=\frac{1}{1+e^{-z}}$$

where *z* is a linear combination of the input variables:

(4) $$z=\beta_{0}+\beta_{1}x_{1}+\beta_{2}x_{2}+\ldots +\beta_{p}x_{p}.$$

The output of the logistic function is always between 0 and 1, making it suitable for probability estimation.

The LR model can be expressed as follows:

(5) $$P\left(y=1|x\right)=\sigma \left(\beta_{0}+\beta_{1}x_{1}+\beta_{2}x_{2}+\ldots +\beta_{p}x_{p}\right),$$

or equivalently,

(6) $$P\left(y=1|x\right)=\frac{1}{1+e^{-(\beta_{0}+\beta_{1}x_{1}+\beta_{2}x_{2}+\ldots +\beta_{p}x_{p})}},$$

where P(y=1|x) is the probability of the outcome *y* being 1, given the predictor variables *x*, and $\beta_{0},\beta_{1},\ldots ,\beta_{p}$ are the coefficients.

LR is a widely used statistical method for binary classification problems. It is a simple yet powerful tool for modeling the relationship between a dependent binary variable and one or more independent variables. Here, we explore the key reasons for its popularity and effectiveness in various applications. One of the primary reasons for using LR is its interpretability. The coefficients of the LR model provide insights into the relationship between the independent variables and the probability of the outcome. The odds ratio, derived from the coefficients, indicates how a one-unit change in an independent variable affects the odds of the outcome occurring. This makes LR particularly useful in fields where understanding the impact of variables is crucial, such as medicine and social sciences. LR models the probability of a binary outcome using the logistic function. This probabilistic output is useful for applications that require not just a classification decision but also a measure of confidence in the prediction. For instance, in credit scoring the probability of default can inform risk management decisions [48].

### 2.7. Artificial Neural Networks

ANNs are computational models inspired by the human brain. They consist of interconnected groups of artificial neurons that process information using a connectionist approach to computation. ANNs are widely used for pattern recognition, classification, regression, and many other applications in ML. The basic unit of an ANN is the neuron, also known as a node or perceptron. A neuron receives one or more inputs, processes them, and produces an output. The fundamental structure of an ANN is as follows [49]:

- **Input Layer:** This layer receives the input signals.
- **Hidden Layers:** These layers perform intermediate computations and feature extraction; there can be one or more hidden layers in an ANN.
- **Output Layer:** This layer produces the final output of the network.

Each neuron in a layer is connected to every neuron in the subsequent layer. The connections have associated weights *w* that are adjusted during training to minimize the error of the network. The output of a neuron is computed as follows:

$$z=\sum_{i=1}^{n}w_{i}x_{i}+b$$

where $x_{i}$ are the inputs, $w_{i}$ are the weights, and *b* is the bias term. The neuron’s activation *a* is then computed using an activation function ϕ:

a=ϕ(z).

Common activation functions include:

- **Sigmoid:**$\;\phi \left(z\right)=\frac{1}{1+e^{-z}}$.
- **Hyperbolic Tangent (Tanh):** ϕ(z)=tanh(z).
- **Rectified Linear Unit (ReLU):** ϕ(z)=max(0,z).
- **Softmax:** Used in the output layer for classification tasks, defined as $\phi \left(z_{i}\right)=\frac{e^{z_{i}}}{\sum_{j}e^{z_{j}}}$.

One of the most significant advantages of ANNs is their ability to capture and model complex nonlinear relationships in data. Traditional linear models may fail to accurately represent such relationships, but ANNs, with their multi-layered structure and nonlinear activation functions, can approximate any continuous function. This makes them highly effective for tasks where the relationship between inputs and outputs is not straightforward. ANNs have demonstrated high predictive performance in a wide range of applications, often outperforming traditional models. Their ability to learn from large amounts of data and generalize well to unseen data makes them suitable for various tasks, such as image and speech recognition, NLP, and time series forecasting. ANNs are highly flexible and can be tailored to solve different types of problems, including classification, regression, clustering, and reinforcement learning. The architecture of an ANN can be adjusted by varying the number of layers, the number of neurons in each layer, and the choice of activation functions, making them adaptable to a wide range of tasks [50].

ANNs can automatically learn and extract relevant features from raw data, reducing the need for manual feature engineering. This is particularly beneficial in fields such as computer vision and NLP where the raw data are high-dimensional and complex. Convolutional Neural Networks, a type of ANN, are specifically designed for image data and can learn hierarchical feature representations through convolutional layers. ANNs are well-suited for handling large datasets with many features. Their distributed computing capabilities, especially when implemented on modern hardware such as GPUs and TPUs, enable them to efficiently process and learn from vast amounts of data. This scalability is crucial for applications such as big data analytics and large-scale industrial processes. Due to their distributed nature, ANNs exhibits robustness and fault tolerance. They can continue to function even if some of their neurons or connections fail, which is analogous to the human brain’s ability to cope with minor damage. This characteristic enhances the reliability of ANN-based systems in critical applications [51].

### 2.8. K-Nearest Neighbors

In this article, we use the R programming language to create a KNN classifier. The Siglo XXI dataset is examined for KNN classifier implementation in R programming language using the caret package [52]. Our goal is to provide predictions of T2DM diagnosis over the patient data.

KNN is one of the simplest ML algorithms, both conceptually and in terms of implementation, and is widely used in the scientific community. The basic idea is straightforward: to classify a new data point, the algorithm identifies the *k* closest data points in the training set and assigns the most common class among those neighbors. Unlike many other ML algorithms, KNN does not require an explicit training phase. It is an instance-based learning algorithm, meaning that it stores all training examples and performs classification only when a new query is made. This can be advantageous in scenarios where the training data can change frequently, as KNN can adapt without needing to be retrained. For comparison with the other models, KNN can be used for both classification and regression tasks. In classification, it predicts the class of a data point based on the majority vote of its neighbors, while in regression it predicts the value of a data point based on the average of its neighbors’ values [53]. KNN is a non-parametric algorithm, meaning that it does not make any assumptions about the underlying data distribution. This is beneficial when dealing with complex and irregular data distributions where parametric methods might fail. KNN can effectively handle such datasets by relying on the local structure of the data, making it a precise tool that can be compared in terms of performance with potent models such as ANN, LR, and SVM [50].

The KNN classifier algorithm works on the principle of finding a predefined K, which is the number of training samples that are closest in distance to a new point, then predicting a label for a given unique point using these samples. The Euclidean distance is the most commonly used distance measure; in this context, the Euclidean distance is often abbreviated as the distance. When dealing with dense or continuous data, Euclidean distance measures are highly recommended, as they represent the best measure of proximity. The KNN classifier is also classified as an instance-based learning/non-generalizing algorithm. It stores training data records in a multidimensional space, recalculates Euclidean distances, and predicts the target class for each new sample and value of K. As a result, it does not generate a generalized internal model [54].

The Euclidean distance is defined by

(7) $$d\left(x_{i},x_{l}\right)=\sqrt{{\left(x_{i1}-x_{l1}\right)}^{2}+{\left(x_{i2}-x_{l2}\right)}^{2}+\cdots +{\left(x_{ip}-x_{lp}\right)}^{2}},$$

where $x_{i}$ is an input sample with *p* features...($x_{i1},x_{i2},\ldots ,x_{ip}$), *n* is the total number of input samples (i=1,2,…,n), and *p* the total number of features (j=1,2,…,p).

In addition, $d(x_{i},x_{l})$ is the Euclidean distance between the points. The *n* Euclidean distances are computed in non-descending order. We take the first *k* distances from this sorted list, as *k* is an integer, then find the k-points that correspond to these k-distances. Finally, we define $k_{i}$ as the number of points in the *i*th class among *k* points.

### 2.9. Nearest Centroid

Nearcent is one of the simplest classifiers; nevertheless, it is capable of classifying data without any feature selection, for example, to find factors in diabetes [55]. In addition, it is extremely fast and requires low computational power, provides a base-line for evaluation of feature selection algorithms, and allows for testing a number of previously inapplicable algorithms. Nearcent and KNN provide similar approaches when there is limited knowledge in the distribution. In this study, they can help to validate each other’s classifications results. Instead of computing distances to all training points, as in KNN, Nearcent only requires distances to the centroids. Because Nearcent uses the mean of all points in a class, it can be more robust to noisy data and outliers compared to KNN. In KNN, the presence of outliers among the neighbors can skew the classification result, whereas in Nearcent, the effect of outliers is averaged out. The Nearcent approach provides a clear and interpretable model by summarizing each class using its centroid [56]. This can be particularly useful for understanding the underlying structure of the data and for explaining the model to stakeholders. KNN can create highly flexible and nonlinear decision boundaries, adapting closely to the data distribution. While this flexibility can lead to better performance in complex datasets, it also makes KNN more prone to overfitting. Nearcent, with its linear decision boundaries, offers a simpler model that can generalize better in certain cases [57].

Nearcent is defined as follows:

(8) $$\vec{\mu_{\ell }}=\frac{1}{|C_{\ell }|}\sum_{i\in C_{\ell }}x_{i}.$$

Given labeled training samples $\left(\vec{x_{1}},y_{1}\right)$, …,$\left(\vec{x_{n}},y_{n}\right)$ with class labels $y_{i}\in Y$, the per-class centroids ${\vec{\mu }}_{\ell }=\frac{1}{|C_{\ell }|}\sum_{i\in C_{\ell }}{\vec{x}}_{i}$ are computed, where $C_{\ell }$ is the set of indices of samples belonging to class ℓ∈Y.

### 2.10. Support Vector Machines

SVM is robust and precise for solving binary classification ML problems. This model uses the theory of Structural Risk Minimization to maximize its prediction accuracy and avoid data overfitting. This model can use a wide variety of kernels, either standard or custom. The radial kernel SVM model used in this study fits the closest observations into the new observation, grouping them in a similar process to KNN based on how much they influence the output of the set classifier for multiple hyperplanes. This kernel has been proven to be one of the most accurate for solving nonlinear separation problems [58].

One of the primary reasons for using SVM is its effectiveness in high-dimensional spaces. SVM can handle datasets with a large number of features, as is the case in this study, making it suitable for applications in bioinformatics. The use of kernels allows SVM to create complex decision boundaries in high-dimensional feature spaces. SVM is designed to maximize the margin between the decision boundary and the nearest data points from any class, known as support vectors. This margin maximization helps SVM achieve a good balance between bias and variance while reducing the risk of overfitting, especially in high-dimensional spaces [56].

SVM is highly flexible due to its use of kernel functions. Kernels allow SVM to model nonlinear relationships by mapping input features into higher-dimensional spaces where a linear decision boundary can separate the classes. Commonly used kernels include:

- Linear Kernel:
  (9) $$K\left(x_{1},x_{2}\right)=x_{1}^{⊤}x_{2}.$$
- Polynomial Kernel:
  (10) $$K\left(x_{1},x_{2}\right)={\left(x_{1}^{⊤}x_{2}+1\right)}^{d}.$$
- Radial Basis Function (RBF) or Gaussian Kernel:
  (11) $$K\left(x_{1},x_{2}\right)=\exp\left(-\frac{∥x_{1}-x_{2}{∥}^{2}}{2\sigma^{2}}\right).$$
- Sigmoid Kernel:
  (12) $$K\left(x_{1},x_{2}\right)=\tanh\left(\beta_{0}x_{1}^{⊤}x_{2}+\beta_{1}\right).$$

### 2.11. Implementation

All models and the methodology were implemented in R version 3.6.3, Copyright (C) 2020 The R Foundation for statistical computing, located in Vienna, Austria. This software is a well-known open source validated by the scientific community; we used the following packages:

- GA was implemented using “galgo 1.4” [41].
- The Generalized Linear Model was implemented with “caret” [52].
- For SVM, “caret” [52] was used.
- The ANN was implemented in R with “caret” [52].
- For KNN, “caret” [52] was used.
- Nearcent was implemented in R with “caret” [52].
- LASSO was implemented using “glmnet” [59].
- The ensemble was implemented in R with nested decisions (use of if).
- RFE was implemented in Python version 3.9.7 by Guido van Rossum at Stichting Mathematisch Centrum in the Netherlands.

## 3. Results

The analyzed data consisted of three datasets (Section 2.1); one called Siglo XXI overall, including 1726 patients, which included 887 cases of T2DM and 839 control subjects, while the other two datasets consisted of separated male and female patients, with the datasets encompassing 855 men and 871 women, respectively. The data treatment (Section 2.2) consisted of data imputation and inclusion criteria (Section 2.3), resulting in balanced data of the three datasets and twelve features discarded. The 21 features were processed in the Siglo XXI overall dataset and 20 in the Male and Female datasets with the feature selection implementations (Section 2.4), obtaining different set of features for the models integrated in the ensemble model.

The metrics were calculated with the following equations:

(13) $$Sensitivity=\frac{T_{p}}{\left(T_{p}+F_{n}\right)}$$

(14) $$Specificity=\frac{T_{n}}{\left(F_{p}+T_{n}\right)}$$

(15) $$Precision=\frac{T_{p}}{\left(T_{p}+F_{p}\right)}$$

(16) $$NegativePredictiveValue=\frac{T_{n}}{\left(T_{n}+F_{n}\right)}$$

(17) $$FalsePositiveRate=\frac{F_{p}}{\left(F_{p}+T_{n}\right)}$$

(18) $$FalseNegativeRate=\frac{F_{n}}{\left(F_{n}+T_{p}\right)}$$

(19) $$Accuracy=\frac{\left(T_{p}+T_{n}\right)}{\left(T_{p}+T_{n}+F_{p}+F_{n}\right)}$$

(20) $$F1Score=\frac{2T_{p}}{\left(2T_{p}+F_{p}+F_{n}\right)}$$

These metrics are described in Table 6, shows which of the implemented models is best for identifying T2DM patients.

The confusion matrix structure is described in Table 7 and contains the following:

- $T_{p}=$ True positive, number of subjects with T2DM correctly classified.
- $F_{p}=$ False positive, number of healthy subjects incorrectly classified.
- $T_{n}=$ True negative, number of healthy subjects correctly classified.
- $F_{n}=$ False negative, number of subjects with T2DM classified as healthy.

### 3.1. Feature Selection Results

The analyzed data consistently provided models with over 0.8 of accuracy for testing by the ensemble, the Summary of all feature selections performed is presented in Table 8. In this blind test performed with low loss compared with the trained model, the model for the blind test metrics is shown in the next subsection.

#### 3.1.1. Features Obtained by GALGO KNN Method

For the features obtained by GALGO and the FS best model, as shown in Table 9, 9 of 21 features were obtained, with an average accuracy of 0.8312.

For the features obtained by GALGO for the male dataset and the FS best model, as shown in Table 10, 11 of 20 features were obtained, with an average accuracy of 0.8714.

For the features obtained by GALGO for the female dataset and the FS best model, as shown in Table 10, 14 of 20 features were obtained, with an average accuracy of 0.8312.

#### 3.1.2. Features Obtained by GALGO Nearcent Method

For the features obtained by GALGO and the FS best model in the overall dataset, as shown in Table 11, 16 of 21 features were obtained, with an average accuracy of 0.8503.

For the features obtained by GALGO for the male dataset and the FS best model, as shown in Table 12, 4 of 20 features were obtained, with an average accuracy of 0.8799.

For the features obtained by GALGO for the female dataset and the FS best model, as shown in Table 12, 7 of 20 features were obtained, with an average accuracy of 0.8503.

#### 3.1.3. Features Obtained by GALGO SVM Method

For the features obtained by GALGO and the FS best model in the overall dataset, as shown in Table 13, 18 of 21 features were obtained, with an average accuracy of 0.8705.

For the features obtained by GALGO for the male dataset and the FS best model, as shown in Table 14, 8 of 20 features were obtained, with an average accuracy of 0.8923.

For the features obtained by GALGO for the female dataset and the FS best model, as shown in Table 14, 9 of 20 features were obtained, with an average accuracy of 0.8364.

#### 3.1.4. Features Obtained by GALGO LR Method

For the features obtained by GALGO and the FS best model in the overall dataset, as shown in Table 15, 18 of 21 features were obtained, with an average accuracy of 0.8652.

For the features obtained by GALGO for the male dataset and the FS best model, as shown in Table 16, 4 of 20 features were obtained, with an average accuracy of 0.883.

For the features obtained by GALGO for the female dataset and the FS best model, as shown in Table 16, 13 of 20 features were obtained, with an average accuracy of 0.8456.

#### 3.1.5. Features Obtained by GALGO NNET Method

For the features obtained by GALGO and the FS best model in the overall dataset, as shown in Table 17, 2 of 21 features were obtained, with an average accuracy of 0.793.

For the features obtained by GALGO for the male dataset and the FS best model, as shown in Table 18, 15 of 20 features were obtained, with an average accuracy of 0.8074.

For the features obtained by GALGO for the female dataset and the FS best model, as shown in Table 18, 13 of 20 features were obtained, with an average accuracy of 0.782.

#### 3.1.6. LASSO

This selector offered a straightforward method and demonstrated high sensitivity in the overall dataset. For the male subset, it achieved the highest specificity and a precision, making it the top-performing model based on precision. The accuracy indicated a robust model capable of competing with predictions made by GA or RFE. The features selected by this technique performed in all datasets are presented in Table 19 and Table 20.

#### 3.1.7. RFE

RFE was implemented with three different selection methods: LR, SVM with linear kernel, and RF. The resulting features of this feature selection technique in the Overall dataset is presented in Table 21, the Male and Female Datasets features resulted are shown in Table 22, Table 23 and Table 24.

### 3.2. Ensemble Model Metrics Results

All the ensemble model results can be accessed in the summary presented in Table 25. Based on the implementation results of the performance metrics for the different models (LR, SVM, and RF), the RF model performed better across multiple measures compared to LR and SVM. RF had the highest sensitivity of 0.8682, indicating better ability to correctly identify positive cases, compared to LR with 0.8127 and SVM with 0.8137. In terms of specificity, RF has the best performance 0.8958, outperforming LR with 0.8640 and with SVM 0.8543, demonstrating a better ability to correctly identify negative cases. RF has the highest precision of 0.8924, indicating a lower rate of false positives compared to LR with 0.8645 and SVM with 0.8526. RF achieved the highest accuracy of 0.8820, suggesting overall superior performance in correctly classifying both positive and negative cases, compared to LR with 0.8375 and SVM with 0.8337. Lastly, RF has the highest F1 score of 0.8802. The F1 score is the harmonic mean of precision and sensitivity, suggesting a better balance between precision and recall compared to LR with 0.8378 and SVM with 0.8327.

The RF model demonstrates superior performance in terms of sensitivity, specificity, precision, accuracy, and F1 score. It seems to be a more effective model for the given task or dataset compared to LR and SVM models in this case. The features contained in RF are “Age”, “Lipids Treatment”, “Triglycerides”, “DBP”, and “DBPU”.

In this case, both the LR and RF models performed equally well across all measures. A sensitivity of 0.8314 indicates equal ability to correctly identify positive cases. SVM performed slightly better in this aspect, with a score of 0.8452. LR and RF outperformed SVM, with specificity of 0.9167 compared to 0.8119, demonstrating better ability to correctly identify negative cases. LR and RF achieved the highest precision of 0.9533, indicating a lower rate of false positives compared to SVM with 0.8733. LR and RF had the same accuracy of 0.8594, higher than SVM at 0.8320, suggesting better overall performance in correctly classifying positive and negative cases. LR and RF had the same F1 score of 0.8882, higher than SVM at 0.8590, indicating a better balance between precision and recall.

Based on the presented metrics, both the LR and RF models performed equally well on the Male dataset, demonstrating high accuracy, sensitivity, specificity, precision, and F1 score. These models seem to be more effective for the given task or dataset compared to the SVM model. The model features for RFE-LR were “Cholesterol”, “TCHOLU”, “SBP”, “DBP”, and “SBPU”, while for RFE-RF they were “Age”, “BMI”, “Lipids Treatment”, “DBP”, and “DBPU”.

On the Female dataset, the LR model appears to perform better overall. LR had the highest sensitivity among the models at 0.7545, indicating a better ability to correctly identify positive cases. SVM with 0.7455 and RF with 0.7333 had slightly lower sensitivities. LR achieved the highest specificity of 0.8800, indicating a better ability to correctly identify negative cases. SVM followed closely at 0.8733, while at 0.7941 RF had a lower specificity. LR had the highest precision of 0.8218, indicating a lower rate of false positives. SVM followed ith 0.8119 and RF had 0.6535. LR had the highest accuracy of 0.8269, indicating better overall performance in correctly classifying both positive and negative cases. SVM followed with 0.8192 and RF with 0.7731. LR had the highest F1 score of 0.7867, indicating effective balancing of precision and recall, while SVM followed with 0.7773 and RF with 0.6911.

The features implemented with RFE-LR in this dataset were “WHR”, “Cholesterol”, “Triglycerides”, “TCHOLU”, “TGU”, “SBP”, “DBP”, and “SBPU”.

All the present analysis in RFE selection and ensemble model implementation is made based in the results shown in Table 26, Table 27 and Table 28.

On the Overall dataset, the LR model appears to perform best. LR has the highest sensitivity of 0.8690, indicating a better ability to correctly identify positive cases. SVM has 0.8511, Nearcent 0.8377, KNN 0.8028, and ANN 0.5781, in descending order. KNN achieved the highest specificity of 0.9167, indicating better ability to correctly identify negative cases. SVM had 0.8902, Nearcent 0.8849, LR 0.8792, and ANN 0.6054, in descending order. KNN had the highest precision of 0.9243, indicating a lower rate of false positives. SVM had 0.8884, Nearcent 0.8845, LR 0.8725, and ANN 0.5896, in descending order. LR had the highest accuracy of 0.8743, indicating better overall performance in correctly classifying positive and negative cases. SVM had 0.8704, Nearcent 0.8607, KNN 0.8530 and ANN 0.5919, in descending order. LR had the highest F1 score of 0.8708, indicating an effective balance of precision and recall. SVM had 0.8694, Nearcent 0.8605, KNN 0.8593, and ANN 0.5838, in descending order.

The LR model outperformed the KNN, Nearcent, SVM, and ANN models with the following features: “Sex”, “Creatinine”, “SBP”, “TGU”, “TCHOLU”, “Cholesterol”, “SBPU”, “LDL”, “LDLU”, “Hypertension Treatment”, “DBP”, “WHR”, “Lipids Treatment”, “HDL”, “Triglycerides”, “HDLU”, “Age”, and “BMI”.

On the Male dataset, the ANN model appears to perform the best overall based on key metrics. ANN had the highest sensitivity of 0.8443, indicating better ability to correctly identify positive cases. KNN (0.8373), SVM (0.8235), LR (0.6859), and Nearcent (0.6856) follow in descending order. ANN achieved the highest specificity of 0.8989, indicating a better ability to correctly identify negative cases. SVM with 0.8837, KNN with 0.8778, LR with 0.7077, and Nearcent with 0.7258 follow in descending order. ANN had the highest precision of 0.9400, indicating a lower rate of false positives. SVM (0.9333), KNN (0.9267), Nearcent (0.8867), and LR (0.8733) follow in descending order. ANN had the highest accuracy of 0.8633, indicating better overall performance in correctly classifying both positive and negative cases. KNN with 0.8516, SVM with 0.8438, Nearcent with 0.6953, and LR with 0.6914 follow in descending order. ANN had the highest F1 score of 0.8896, indicating an effective balance of precision and recall. KNN (0.8797), SVM (0.8750), Nearcent (0.7733), and LR (0.7683) follow in descending order.

The ANN model outperforms the KNN, Nearcent, SVM, and LR models on the Male dataset with these features: “Creatinine”, “SBP”, “TGU”, “SBPU”, “TCHOLU”, “Cholesterol”, “BMI”, “Hypertension Treatment”, “Urea”, “DBP”, “Age”, “LDL”, “HDL”, “HDLU”, and “LDLU”.

On the Female dataset, the LR model appears to perform the best overall. LR had the highest sensitivity of 0.7719, indicating better ability to correctly identify positive cases. KNN with 0.7658, ANN with 0.7748, SVM with 0.7000, and Nearcent with 0.6804 follow in descending order. LR achieved the highest specificityof 0.9110, indicating a better ability to correctly identify negative cases. ANN with 0.8993, KNN with 0.8926, SVM with 0.8786, and Nearcent with 0.7853 follow in descending order. LR had the highest precision of 0.8713, indicating a lower rate of false positives. ANN with 0.8515, KNN with 0.8416, SVM with 0.8317, and Nearcent with 0.6535 follow in descending order. At 0.8500, LR had the highest accuracy, indicating better overall performance in correctly classifying both positive and negative cases. ANN with 0.8462, KNN with 0.8385, SVM with 0.7962, and Nearcent with 0.7462 follow in descending order. LR had the highest F1 score 0.8186, effectively balancing precision and recall. ANN with 0.8113, KNN with 0.8019, SVM with 0.7602, and Nearcent with 0.6667 follow in descending order.

The LR model outperforms the KNN, Nearcent, SVM, and ANN models on the Female dataset with these features: “TGU”, “Creatinine”, “WHR”, “LDL”, “LDLU”, “SBP”, “SBPU”, “TCHOLU”, “Cholesterol”, “BMI”, “Lipids Treatment”, “Triglycerides”, and “Age”.

All the present analysis in GALGO selection and ensemble model implementation is made based in the results shown in Table 29, Table 30 and Table 31.

The ensemble model with LASSO feature selection performed well across all datasets, with particularly notable performance on the Male dataset.

For the Overall dataset, the model showed balanced performance, with relatively high sensitivity, specificity, precision, accuracy and F1 score. It performed well in identifying both positive and negative cases. The model performed exceptionally well on the Male dataset, especially in terms of sensitivity and precision, showing a high ability to correctly identify positive cases (sensitivity) and low rate of false positives (precision). The model had lower sensitivity and precision on the Female dataset compared to the Male dataset, resulting in a slightly lower overall accuracy and F1 score. It may be worth exploring ways to improve performance on the Female dataset specifically.

The Male dataset LASSO implementation had the following features: “Age”, “WHR”, “Urea”, “Lipids Treatment”, “HDL”, “Triglycerides”, “Hypertension Treatment”, “DBP”, and “SBPU”.

All the present analysis in LASSO selection and ensemble model implementation is made based in the results shown in Table 32.

Calculate the AIC requires the log-likelihood of the model and the number of parameters.

Assuming that the log-likelihood values and number of parameters are available for each model, the formula is as follows:

$$AICc=-2\times \text{Log-Likelihood}+2k\times \frac{n}{n-k-1}$$

where:

- Log-Likelihood is the maximized value of the likelihood function,
- *k* is the number of parameters in the model,
- *n* is the number of observations.

These sets were selected by lowest AICc implemented in the ensemble: Overall dataset RFE-RF: “Age”, “Lipids Treatment”, “Triglycerides”, “DBP”, and “DBPU”, with an accuracy of 0.8820. Male dataset LASSO: “Age”, “WHR”, “Urea”, “Lipids Treatment”, “HDL”, “Triglycerides”, “Hypertension Treatment”, “DBP”, and “SBPU”, with an accuracy of 0.8633. Female dataset RFE-LR: “WHR”, “Cholesterol”, “Triglycerides”, “TCHOLU”, “TGU”, “SBP”, “DBP”, and “SBPU”, with an accuracy of 0.8269.

### 3.3. Ranges

The ranges presented in this section serve as a foundation for establishing normal physiological variations and identifying potential outliers or abnormal values within different datasets. All ranges can be accessed in the Summary Table 33. Understanding these ranges is essential in order for clinicians, researchers, and healthcare practitioners to interpret health assessments while making informed diagnostic decisions and tailoring interventions to individualized patient needs. The comprehensive nature of these ranges ensures a thorough characterization of the health indicators under investigation.

The age range represents the span of ages observed within each dataset. It includes the minimum and maximum values as well as quartiles, providing insights into the central tendency and dispersion of age among cases. WHR ranges encompass the variability in the ratio of waist circumference to hip circumference. Quartiles and extreme values offer a comprehensive view of the distribution of this anthropometric measures seen in the data, which is crucial for assessing body fat distribution. BMI ranges depict the distribution of body mass indices within each dataset. They include quartiles and extreme values, allowing for an understanding of the variability in body weight relative to height among different cases. Ranges for urea and creatinine levels provide insights into the distribution of these renal function indicators. Minimum, maximum, and quartile values offer a comprehensive overview of the variability in these biochemical markers. Cholesterol profile ranges encompass total cholesterol, HDL cholesterol, LDL cholesterol, and triglyceride levels. These ranges offer a detailed perspective on the distribution of lipid markers, which is crucial for cardiovascular health assessments. Blood pressure ranges include systolic (SBP, SBPU) and diastolic (DBP, DBPU) values. Quartiles and extreme values provide a comprehensive understanding of the variability in blood pressure within each dataset, which is crucial for cardiovascular health assessment.

Each table represents a specific dataset (Overall in Table 34, Male in Table 35 and Female in Table 36) with controls (Overall in Table 37, Male in Table 38 and Female in Table 39) and cases(Overall in Table 40, Male in Table 41 and Female in Table 42); the ranges of the variables differ among these datasets. For instance, the Male dataset tends to have higher values for variables such as BMI, Creatinine and LDL compared to the female dataset The Male and Female datasets include sex-specific variables such as TCHOLU, HDLU, LDLU, and TGU, which are related to cholesterol levels in urea. These variables are not present in the Overall dataset. Blood pressure variables (SBP, SBPU, DBP, DBPU) exhibit variability across the datasets, reflecting sex-related differences in blood pressure distributions. The Female dataset generally exhibits higher values for CHOL, HDLc, LDL, Triglycerides, and TCHOLU compared to the Male dataset. The age distribution varies across the datasets, with the Male dataset having slightly higher median and upper quartile values compared to the Female dataset. Finally, the Male dataset tends to have a slightly higher WHR compared to the Female dataset.

The SBP and DBP values for Controls only follow a similar pattern across the Overall, Male, and Female datasets, with overlapping ranges for SBP, SBPU, DBP, and DBPU. While the minimum and maximum ages are consistent across all datasets, there are subtle differences in the median and upper quartile values. The Female dataset generally exhibits a slightly higher median and upper quartile age compared to the Male dataset. WHR values vary among the datasets, with the Female dataset showing a lower median and upper quartile compared to the Male dataset. BMI ranges differ, with the Female dataset tending to have lower median and upper quartile values compared to the Male dataset. The Overall dataset falls in between the Male and Female datasets regarding BMI. Variability exists in cholesterol and lipid profiles, with the Female dataset generally exhibiting lower values for CHOL, HDL, LDL, Triglycerides, and TCHOLU compared to the Male dataset. Creatinine levels, represented by Creatinine, vary among the datasets, with the Female dataset showing slightly lower median and upper quartile values compared to the Male dataset.

Lastly, for Cases only, the minimum and maximum age values are consistent across all datasets, indicating a uniform age distribution within cases. Cases presenting Systolic (SBP, SBPU) and diastolic (DBP, DBPU) blood pressure values exhibit comparable patterns across the Overall, Male, and Female datasets. Urea and creatinine levels show similar patterns among the datasets, with overlapping ranges. Total cholesterol, HDL cholesterol, LDL cholesterol, and Triglycerides follow similar distribution trends across the Male, Female, and the Overall datasets. WHR values vary among the datasets, with the Female dataset generally exhibiting lower median and upper quartile values compared to the Male dataset. BMI ranges differ, with the Female dataset tending to have lower median and upper quartile values compared to the Male dataset. The Overall dataset falls in between the Male and Female datasets regarding BMI. Creatinine levels in the Female dataset show slightly lower median and upper quartile values compared to the Male dataset. Finally, variability exists in total cholesterol, with the Female dataset generally exhibiting higher values compared to the Male dataset.

## 4. Discussion

The distribution and variability of various health-related metrics across different datasets, including the Overall, Male, and Female datasets for both controls and cases, include variables such as TCHOLU, HDLU, LDLU, and TGU related to cholesterol levels in urea that are present in the Male and Female datasets but not in the Overall dataset. The inclusion of these variables in the sex-specific datasets indicates a focus on sex-related variations in cholesterol levels [60], potentially emphasizing the importance of these variables in understanding sex-specific health risks planning safety over prescriptions or oral medication [61]. Blood pressure variables (SBP, SBPU, DBP, DBPU) exhibit variability across datasets, reflecting sex-related differences in blood pressure distributions. The consistency in SBP and DBP patterns across the Overall, Male, and Female datasets suggests that blood pressure trends remain relatively similar regardless of sex [62]. While the minimum and maximum ages are consistent across all datasets, there are subtle differences in the median and upper quartile values. The Female dataset generally exhibits slightly higher median and upper quartile age compared to the Male dataset, suggesting potential age-related differences between males and females.

WHR and BMI values vary among datasets, with the Female dataset generally showing lower median and upper quartile values compared to the Male dataset. These differences highlight a tendency for potential sex-related variations in body composition to affect energy balance and increase the risk of developing IR and obesity-related complications [63], with females tending to have lower WHR and BMI compared to males. Variability exists in cholesterol and lipid profiles, with the Female dataset generally exhibiting lower values for CHOL, HDL, LDL, Triglycerides and TCHOLU compared to the Male dataset. This comparison is aligned to look for key factors in T2DM comorbidities and cardiovascular diseases [64]. This sex-related variability in lipid profiles emphasizes the importance of considering sex-specific differences in cardiovascular risk factors. Creatinine levels vary among datasets, with the Female dataset showing slightly lower median and upper quartile values compared to the Male dataset, providing useful information to find risk factors for DKD. This difference may reflect sex-related variations in renal function or muscle mass [65]. The cases exhibit a uniform age distribution across all datasets, indicating consistency in age representation within cases. This uniformity simplifies age-related comparisons and analyses within cases. In the cases, SBP and DBP values as well as cholesterol and lipid profiles exhibit comparable patterns across the Male, Female, and Overall datasets. This consistency suggests that certain health metrics maintain similar trends across different datasets when considering cases.

This analysis provides valuable insights into the variations and distributions of health-related metrics across different datasets, highlighting potential sex-related differences and consistent patterns within cases. These findings can guide further investigations into the underlying factors contributing to these variations and inform personalized healthcare strategies.

To determine the importance of “Triglycerides” and “DBP” in the overall dataset, we analyzed their ranges and significance in the context of health and clinical relevance. It its known that elevated levels of triglycerides are associated with an increased risk of cardiovascular diseases, while triglycerides are crucial for assessing lipid metabolism and overall cardiovascular health. The range of triglycerides in the dataset spans from 32 to 371, indicating considerable variability in triglyceride levels. Higher values, especially beyond the upper quartile, may suggest potential cardiovascular risk and metabolic abnormalities. DBP is a key component in evaluating blood pressure, which is a critical indicator of cardiovascular health. Elevated DBP is associated with an increased risk of heart disease and other cardiovascular events. The range of DBP in the dataset varies from 50 to 105, covering a spectrum of blood pressure levels. Higher DBP values may indicate hypertension and increased cardiovascular risk.

Both “Triglycerides” and “DBP” are vital indicators of cardiovascular health, and are commonly used in clinical assessments. The wide ranges observed in the dataset emphasize the diversity of health conditions among individuals. Monitoring these parameters is crucial for identifying potential health issues, especially in the context of cardiovascular diseases. In the overall dataset, “Triglycerides” and “DBP” stand out as important features due to their clinical significance in assessing cardiovascular health as comorbidities or diseases directly related to T2DM. Monitoring and managing triglyceride levels and blood pressure, especially diastolic pressure, is essential for overall health and prevention of cardiovascular diseases.

The range of triglycerides in controls spanned from 32 to 299, indicating considerable variability in triglyceride levels among individuals without T2DM. Elevated triglyceride levels, especially beyond the upper quartile, may suggest potential cardiovascular risk even in the absence of diabetes. The range of triglycerides in the cases spans from 41.00 to 417.10, indicating higher levels, especially beyond the mean, suggesting increased cardiovascular risk and complications in individuals with T2DM.

The range of DBP among the controls in the Overall dataset varied from 46 to 100, covering a spectrum of blood pressure levels in individuals without T2DM. The range of DBP among the cases varied from 65 to 105, showing higher DBP values than the controls, especially beyond the upper quartile, which may indicate hypertension and increased cardiovascular risk in individuals with T2DM.

There are different approaches to differentiating males and females in studies (see related work in Table 43), with unique physical structure, anthropometry, and clinical levels on almost every feature that is measured. Non-invasive biomarkers can be a fast and low cost approach that provides insight into T2DM in a large population with almost no medical intervention [66]. Non-fasting blood tests can also provide a perspective on how metabolomic disorders affect each sex regardless of the patient’s current health, as shown by the study of Wannamethee et al. [67]. The results analyzed by Farran et al. combined four microRNAs (miR-146a-5p, miR-16-2-3p, miR-126-5p, and miR-30d) to distinguish early complications of T2DM, reaching 0.77 accuracy and 0.79 sensitivity. In male patients only, the same microRNAs with the addition of miR-423 could distinguish early complications of T2DM with 0.83 accuracy [68]. Additionally, another study by Akash et al. found that the BMI of female diabetic patients was more strongly correlated with increased incidence of IR compared to male diabetic patients. The key findings of their study indicate that sex differences significantly influence the association of inflammatory biomarkers with the development of IR in T2DM [69].

Age is a significant risk factor for T2DM. As people age, their risk of developing T2DM increases due to factors such as decreased insulin sensitivity and impaired pancreatic beta cell function [40]. Lipid-lowering treatments such as statins are often used in patients who have T2DM or are at risk in order to manage dyslipidemia, which is common in diabetic patients and can contribute to cardiovascular complications [60]. Elevated triglyceride levels are often associated with IR, a hallmark of T2DM; high triglycerides can also indicate metabolic syndrome, which is closely linked to the development of T2DM [40]. Hypertension (high blood pressure) is commonly seen in individuals with T2DM. High DBP specifically can indicate increased cardiovascular risk, and is often seen alongside IR [60]. DBPU could be a measure related to underlying DBP trends or baseline measurements, providing insights into chronic blood pressure management and its impact on T2DM. WHR is a measure of central obesity, which is a major risk factor for T2DM. Central adiposity is closely linked to IR and metabolic syndrome [40]. Elevated blood urea levels can be indicative of kidney dysfunction, which is a common complication of T2DM. Poor kidney function can exacerbate diabetes management and outcomes. Low levels of HDL cholesterol are a risk factor for cardiovascular disease, and are often seen in individuals with T2DM. HDL helps in the removal of cholesterol from the bloodstream, and its deficiency can lead to atherogenic changes [40]. Treatment for hypertension is often necessary in T2DM patients due to the high prevalence of co-occurring hypertension [60]. Managing blood pressure is crucial to reducing cardiovascular risks in diabetics. SBPU could indicate underlying or baseline SBP levels, providing insights into long-term blood pressure control and its impact on T2DM. Total cholesterol levels, including both LDL (bad cholesterol) and HDL, are important in assessing cardiovascular risk. Dyslipidemia is commonly seen in T2DM, and managing cholesterol levels is crucial. TCHOLU could refer to baseline or underlying levels of total cholesterol, helping to understand long-term lipid management and its effects on T2DM progression [40]. Similar to triglycerides, underlying or baseline triglyceride levels can provide insights into long-term lipid management and its impact on metabolic health. High SBP is a common comorbidity in T2DM patients, and is associated with increased cardiovascular risk. Managing SBP is essential for reducing complications in diabetics. Triglycerides, DBP, and SBP are repeatedly selected across different models and datasets, underscoring their significant roles in the pathogenesis and management of T2DM. Elevated triglycerides and blood pressure (both diastolic and systolic) are major risk factors for cardiovascular diseases, which are common complications of T2DM [60].

## 5. Conclusions

The comprehensive analysis of health-related metrics across different datasets, including Overall, Male, and Female datasets, provides valuable insights into sex-related differences and consistent patterns within cases. Sex-related differences in cholesterol and lipid profiles present sex-related variations in variables such as TCHOLU, HDLU, LDLU, and TGU, emphasizing the importance of considering sex-specific differences in cholesterol levels. Females generally exhibit lower cholesterol and lipid values compared to males, highlighting potential sex-related variations in cardiovascular risk factors. Blood pressure and lipid profiles, including SBP and DBP values as well as cholesterol and lipid profiles, exhibit comparable patterns across males, females, and the overall population in cases. This consistency suggests that certain health metrics maintain similar trends across different datasets when considering cases. The significance of triglycerides and DBP as core features (present in all feature selection final methods) have wide ranges in controls and cases, indicating significant variability in triglyceride levels. Elevated levels, especially beyond the upper quartile, may suggest potential cardiovascular risk, emphasizing the importance of monitoring triglyceride levels for overall health. The same is the case with DBP, with a diverse range of DBP values in both controls and cases underscoring its importance in assessing cardiovascular health. Higher DBP values, especially beyond the upper quartile, may indicate hypertension and increased cardiovascular risk.

The clinical significance and implications of triglycerides and DBP in the Overall dataset show that both triglycerides and DBP emerge as crucial indicators of cardiovascular health. Their wide ranges emphasize the diversity of health conditions among individuals, highlighting their clinical significance in assessing cardiovascular health, especially in the context of comorbidities associated with T2DM. Triglycerides in controls exhibit considerable variability in levels, indicating potential cardiovascular risk even in the absence of T2DM. In the cases, higher levels suggest increased cardiovascular risk and complications in individuals with T2DM. Finally, the DBP in cases shows higher values than in controls, particularly beyond the upper quartile, indicating hypertension and increased cardiovascular risk in individuals with T2DM. Understanding these sex-related differences and similarities is crucial for tailoring healthcare strategies and interventions. Monitoring triglyceride levels and blood pressure, especially DBP, is essential for overall health and the prevention of cardiovascular diseases, particularly in the context of T2DM. These findings provide a foundation for further investigations into the underlying factors contributing to these variations and support for the development of personalized healthcare approaches.

This study highlights the need for comprehensive datasets from diverse populations to enhance the generalizability of our findings. While our current sample size is small, we are committed to expanding this research in future work by incorporating more robust datasets, particularly from Latino populations, where there is a noticeable lack of information and experimental approaches. We also highlight the importance of sex-specific biomarkers from other studies, along with additional comparisons across various populations. Understanding the biological mechanisms underlying the identified biomarkers is crucial, as is the collaboration with clinicians and healthcare providers that are essential for translating research findings into real-world clinical practice. However, while this aspect of the present research extends beyond the scope of this paper, we have established groundwork for future studies aimed at developing specific strategies for implementation. This future research will be critical in bridging the gap between our findings and their practical application in clinical settings. 

## Figures and Tables

**Table 1:** Eliminated features.

Feature	Description
plate_info	Hospital patient id
id	Consecutive identification number in dataset
Edu	Education Level
Sal	Salary
Age DX (cases)	Years with diabetes disease diagnosticated
GLU (mg/dL)	Glucose levels
HbA1c	Glycosylated hemoglobin
GLIBENCLAMIDA	If patient has Glibenclamide treatment
GLIBEN_MG_DIA	Glibenclamide prescribed in milligrams
METFORMINA	If patient has Metformin treatment
METFOR_MG_DIA	Metformin prescribed in milligrams
PIOGLITAZONA	If patient has Pioglitazone treatment
PIOGLI_MG_DIA	Pioglitazone prescribed in milligrams
ROSIGLITAZONA	If patient has Rosiglitazone treatment
ROSIGLI_MG_DIA	Rosiglitazone prescribed in milligrams
ACARBOSA	If patient has Acarbose treatment
ACARBO_MG_DIA	Acarbose prescribed in milligrams
INSULINA	If patient has Insulin treatment
INSUL_UI_DIA	Insulin prescribed in milligrams
TIPO_COMPLICACION DE DT2	T2DM complications

**Table 2:** Inclusion criteria.

1. Patients must be at least 18 years old.
2. There must be no differentiation on the data obtained based on sex,
education, ethnicity, race, or marital status.
3. The datasets should exclusively comprise anthropometric and clinic
data for each individual.
4. The dataset should be capable of distinguishing control
subjects from those with T2DM
5. Every subject’s dataset must include complete information
for all features.
6. The data does not contain negative values
7. It does not contain glucose related features

**Table 3:** Features.

Feature	Description
Sex	Patients sex
Age	Age of the patient in years
WHR	Waist to Hip Ratio
BMI	Body Mass Index
Urea	Waste product resulting from the breakdown of protein in the patient body
Creatinine	Waste product produced by muscles as part of regular daily activity
Lipids treatment	Lipid levels in treatment
Cholesterol	Fat-like substance that is found in all cells of the patient body
HDL	High Density Lipoprotein (corrected by medication)
LDL	Low Density Lipoprotein (corrected by medication)
Triglycerides	Type of fat found in the patient body
TCHOLU	Total Cholesterol (uncorrected by medication)
HDLU	High Density Lipoprotein (uncorrected by medication)
LDLU	Low Density Lipoprotein (uncorrected by medication)
TGU	Triglycerides (uncorrected by medication)
SBP	Systolic Blood Pressure (corrected by medication)
DBP	Diastolic Blood Pressure (corrected by medication)
SBPU	Systolic Blood Pressure (uncorrected by medication)
DBPU	Diastolic Blood Pressure (uncorrected by medication)
HA-TX	Subject under Hypertension Treatment
LIPIDS-TX	Subject under Lipids Treatment

**Table 4:** GALGO parameters from the Siglo XXI overall dataset.

Model	Parameter	Value
KNN	classification.method	‘knn’
	chromosomeSize	5
	maxSolutions	2000
	maxGenerations	60
	goalFitness	0.9
Nearcent	classification.method	‘nearcent’
	chromosomeSize	5
	maxSolutions	2000
	maxGenerations	60
	goalFitness	0.9
Artificial Neural Network	classification.method	‘nnet’
	chromosomeSize	5
	maxSolutions	2000
	maxGenerations	60
	goalFitness	0.9
Logistic Regression	classification.method	‘user’
	classification.userFitnessFunc	logreg.R.predict
	chromosomeSize	5
	maxSolutions	2000
	maxGenerations	60
	goalFitness	0.9
Support Vector Machines	classification.method	‘svm’
	svm.kernel	‘radial’
	chromosomeSize	5
	maxSolutions	2000
	maxGenerations	60
	goalFitness	0.9

**Table 5:** GALGO parameters for Siglo XXI male/female datasets.

Model	Parameter	Value
KNN	classification.method	‘knn’
	chromosomeSize	5
	maxSolutions	1600
	maxGenerations	60
	goalFitness	0.9
Nearcent	classification.method	‘nearcent’
	chromosomeSize	5
	maxSolutions	1600
	maxGenerations	60
	goalFitness	0.9
Artificial Neural Network	classification.method	‘nnet’
	chromosomeSize	5
	maxSolutions	1600
	maxGenerations	60
	goalFitness	0.9
Logistic Regression	classification.method	‘user’
	classification.userFitnessFunc	logreg.R.predict
	chromosomeSize	5
	maxSolutions	1600
	maxGenerations	60
	goalFitness	0.9
Support Vector Machines	classification.method	‘svm’
	svm.kernel	‘radial’
	chromosomeSize	5
	maxSolutions	1600
	maxGenerations	60
	goalFitness	0.9

**Table 6:** All metrics in this table were extracted in each model to evaluate performance.

Metric	Description
Sensitivity	Correct identification of patients with T2DM (True Positive).
Specificity	Correct identification of patients without T2DM (True Negative).
Precision	Defines what portion of the positive cases
	of T2DM are actually positive.
Negative Predictive Value	Defines what portion of the negative cases
	of T2DM are actually negative.
False Positive Rate	The rate of the predicted false values that are actually true.
False Negative Rate	The rate of the predicted true values that are actually false.
Accuracy	The percentage of cases that the model has classified correctly.
F1 Score	The measure of precision that a test has.

**Table 7:** Confusion matrix structure.

True Values	Predicted (True)	Predicted (False)
True	$T_{p}$	$T_{n}$
False	$F_{p}$	$F_{n}$

**Table 8:** Summary feature selection.

Technique	Kernel	Dataset	Table
GALGO	KNN	Overall	Table 9
		Male/Female	Table 10
	Nearcent	Overall	Table 11
		Male/Female	Table 12
	SVM	Overall	Table 13
		Male/Female	Table 14
	LR	Overall	Table 15
		Male/Female	Table 16
	NNET	Overall	Table 17
		Male/Female	Table 18
LASSO		Overall	Table 19
		Male/Female	Table 20
RFE	LR, SVM and RF	Overall	Table 21
	LR	Male/Female	Table 22
	SVM	Male/Female	Table 23
	RF	Male/Female	Table 24

**Table 9:** KNN GALGO Siglo XXI overall result features.

Siglo XXI Overall
“Creatinine”, “TGU”, “TCHOLU”, “Sex”, “WHR”, “SBP”, “SBPU”, “Cholesterol” and “Urea”

All of the features in this table were obtained using GALGO and the KNN method, with 2000 Big Bangs and 60 generations.

**Table 10:** KNN GALGO male/female result features.

Siglo XXI Male Dataset	Siglo XXI Female Dataset
“Creatinine”	“Creatinine”
“SBP”	“TGU”
“DBP”	“SBP”
“SBPU”	“WHR”
“Age”	“Cholesterol”
“TGU”	“SBPU”
“BMI”	“BMI”
“Hypertension Treatment”	“Urea”
“HDLU”	“TCHOLU”
“Urea”	“Age”
“TCHOLU”	“LDLU”
	“Hypertension Treatment”
	“LDL”
	“Triglycerides”

All of the features in this table were obtained using GALGO and the KNN method, with 1600 Big Bangs and 60 generations.

**Table 11:** Nearcent GALGO Siglo XXI overall result features.

Siglo XXI Overall
“Creatinine”, “TGU”, “Sex”, “SBPU”, “LDL”, “SBP”, “BMI”,
“WHR”, “Age”, “Triglycerides”, “Cholesterol”, “LDLU”, “Urea”,
“TCHOLU”, “HDLU” and “Lipids Treatment”

All of the features in this table were obtained using GALGO and the Nearcent method, with 2000 Big Bangs and 60 generations.

**Table 12:** Nearcent GALGO male/female result features.

Siglo XXI Male Dataset	Siglo XXI Female Dataset
“Creatinine”	“Creatinine”
“TGU”	“TGU”
“Cholesterol”	“LDL”
“SBP”	“SBP”
	“BMI”
	“SBPU”
	“Cholesterol”

All of the features in this table were obtained using GALGO and the Nearcent method, with 1600 Big Bangs and 60 generations.

**Table 13:** SVM GALGO Siglo XXI overall result features.

Siglo XXI Overall
“Sex”, “Creatinine”, “TGU”, “SBP”, “LDLU”, “SBPU”, “LDL”, “TCHOLU”,
“Cholesterol”, “BMI”, “Age”, “WHR”, “Urea”,
“Lipids Treatment”, “HDL”, “Triglycerides”, “HDLU” and “Hypertension Treatment”

All of the features in this table were obtained using GALGO and the SVM method, with 2000 Big Bangs and 60 generations.

**Table 14:** SVM GALGO male/female result features.

Siglo XXI Male Dataset	Siglo XXI Female Dataset
“Creatinine”	“Creatinine”
“TGU”	“TGU”
“SBPU”	“SBP”
“SBP”	“LDL”
“Age”	“LDLU”
“Cholesterol”	“SBPU”
“TCHOLU”	“BMI”
“BMI”	“TCHOLU”
	“Cholesterol”

All of the features in this table were obtained using GALGO and the SVM method, with 1600 Big Bangs and 60 generations.

**Table 15:** LR GALGO Siglo XXI overall result features.

Siglo XXI Overall
“Sex”, “Creatinine”, “SBP”, “TGU”, “TCHOLU”, “Cholesterol”, “SBPU”,
“LDL”, “LDLU”, “Hypertension Treatment”, “DBP”, “WHR”,
“Lipids Treatment”, “HDL”, “Triglycerides”, “HDLU”, “Age” and “BMI”

All of the features in this table were obtained using GALGO and the LR method, with 2000 Big Bangs and 60 generations.

**Table 16:** LR GALGO male/female result features.

Siglo XXI Male Dataset	Siglo XXI Female Dataset
“SBP”,	“TGU”
“Creatinine”	“Creatinine”
“TGU”	“WHR”
“Cholesterol”	“LDL”
	“LDLU”
	“SBP”
	“SBPU”
	“TCHOLU”
	“Cholesterol”
	“BMI”
	“Lipids Treatment”
	“Triglycerides”
	“Age”

All of the features in this table were obtained using GALGO and the LR method, with 1600 Big Bangs and 60 generations.

**Table 17:** NNET GALGO Siglo XXI overall result features.

Siglo XXI Overall
“Creatinine” and “TGU”

All of the features in this table were obtained using GALGO and the NNET method, with 2000 Big Bangs and 60 generations.

**Table 18:** NNET GALGO male/female result features.

Siglo XXI Male Dataset	Siglo XXI Female Dataset
“Creatinine”	“TGU”
“SBP”	“Creatinine”
“TGU”	“SBP”
“SBPU”	“WHR”
“TCHOLU”	“LDLU”
“Cholesterol”	“LDL”
“BMI”	“SBPU”
“Hypertension Treatment”	“Cholesterol”
“Urea”	“BMI”
“DBP”	“TCHOLU”
“Age”	“Age”
“LDL”	“Lipids Treatment”
“HDL”	“Triglycerides”
“HDLU”	
“LDLU”	

All of the features in this table were obtained using GALGO and the NNET method, with 1600 Big Bangs and 60 generations.

**Table 19:** LASSO Siglo XXI overall result features.

Siglo XXI Overall
“Sex”, “Age”, “WHR”, “BMI”, “Urea”, “Lipids Treatment”,
“HDL”, “Triglycerides”, “Hypertension Treatment”, “DBP” and “SBPU”

All of the features in this table were obtained using LASSO Elastic net method on the Overall dataset.

**Table 20:** LASSO male/female result features.

Siglo XXI Male Dataset	Siglo XXI Female Dataset
“Age”	“Age”
“WHR”	“BMI”
“Urea”	“Urea”
“Lipids Treatment”	“Lipids Treatment”
“HDL”	“HDL”
“Triglycerides”	“Triglycerides”
“Hypertension Treatment”	“Hypertension Treatment”
“DBP”	“DBP”
“SBPU”	

All of the features in this table were obtained using LASSO Elastic net method on the male and female datasets.

**Table 21:** RFE Siglo XXI overall result features with LR, SVM, and RF.

	Siglo XXI Overall	
LR	SVM	RF
“Sex”	“Sex”	“Age”
“WHR”	“WHR”	“Lipids Treatment”
“Cholesterol”	“Creatinine”	“Triglycerides”
“TCHOLU”	“Cholesterol”	“DBP”
“SBP”	“SBP”	“DBP”
“Hypertension Treatment”	“TCHOLU”	
“DBP”	“DBP”	
“SBPU”	“SBPU”	
“DBPU”	“DBPU”	

All of the features in this table were obtained using RFE with LR, SVM and RF as estimators on the Overall dataset.

**Table 22:** RFE male/female LR result features.

Siglo XXI Male Dataset	Siglo XXI Female Dataset
“Cholesterol”	“WHR”
“TCHOLU”	“Cholesterol”
“SBP”	“Triglycerides”
“DBP”	“TCHOLU”
“SBPU”	“TGU”
	“SBP”
	“DBP”
	“SBPU”

All of the features in this table were obtained using RFE with LR as estimator on the male and female dataset.

**Table 23:** RFE male/female SVM result features.

Siglo XXI Male Dataset	Siglo XXI Female Dataset
“Age”	“Age”
“WHR”	“WHR”
“Creatinine”	“BMI”
“SBP”	“Creatinine”
“DBP”	“Cholesterol”
“SBPU”	“TCHOLU”
“DBPU”	“SBP”
	“DBP”
	“SBPU”
	“DBPU”

All of the features in this table were obtained using RFE with SVM with linear kernel as estimator on the male and female dataset.

**Table 24:** RFE male/female RF result features.

Siglo XXI Male Dataset	Siglo XXI Female Dataset
“Age”	“Age”
“BMI”	“WHR”
“Lipids Treatment”	“BMI”
“DBP”	“Triglycerides”
“DBPU”	“DBP”

All of the features in this table were obtained using RFE with RF as estimator on the male and female dataset.

**Table 25:** Summary of ensemble model result metrics.

Model	Dataset	Table
	Overall	Table 26
RFE	Male	Table 27
	Female	Table 28
	Overall	Table 29
GALGO	Male	Table 30
	Female	Table 31
LASSO	Overall/Male/Female	Table 32

**Table 26:** Ensemble model RFE feature selection in Siglo XXI dataset: overall confusion matrix blind test measure values.

Measure	LR	SVM	RF
Sensitivity	0.8127	0.8137	0.8682
Specificity	0.8640	0.8543	0.8958
Precision	0.8645	0.8526	0.8924
Negative Predictive Value	0.8120	0.8158	0.8722
False Positive Rate	0.1360	0.1457	0.1042
False Discovery Rate	0.1355	0.1474	0.1076
False Negative Rate	0.1873	0.1863	0.1318
Accuracy	0.8375	0.8337	0.8820
F1 Score	0.8378	0.8327	0.8802

**Table 27:** Ensemble model RFE feature selection in Siglo XXI Male dataset: confusion matrix blind test measure values.

Measure	LR	SVM	RF
Sensitivity	0.8314	0.8452	0.8314
Specificity	0.9167	0.8119	0.9167
Precision	0.9533	0.8733	0.9533
Negative Predictive Value	0.7264	0.7736	0.7264
False Positive Rate	0.0833	0.1881	0.0833
False Discovery Rate	0.0467	0.1267	0.0467
False Negative Rate	0.1686	0.1548	0.1686
Accuracy	0.8594	0.8320	0.8594
F1 Score	0.8882	0.8590	0.8882

**Table 28:** Ensemble model RFE feature selection in Siglo XXI Female dataset: confusion matrix blind test measure values.

Measure	LR	SVM	RF
Sensitivity	0.7545	0.7455	0.7333
Specificity	0.8800	0.8733	0.7941
Precision	0.8218	0.8119	0.6535
Negative Predictive Value	0.8302	0.8239	0.8491
False Positive Rate	0.1200	0.1267	0.2059
False Discovery Rate	0.1782	0.1881	0.3465
False Negative Rate	0.2455	0.2545	0.2667
Accuracy	0.8269	0.8192	0.7731
F1 Score	0.7867	0.7773	0.6911

**Table 29:** Ensemble model GALGO feature selection in Siglo XXI Overall dataset: confusion matrix blind test measure values.

Measure	KNN	Nearcent	LR	SVM	NN
Sensitivity	0.8028	0.8377	0.8690	0.8511	0.5781
Specificity	0.9167	0.8849	0.8792	0.8902	0.6054
Precision	0.9243	0.8845	0.8725	0.8884	0.5896
Negative Predictive Value	0.7857	0.8383	0.8759	0.8534	0.5940
False Positive Rate	0.0833	0.1151	0.1208	0.1098	0.3946
False Discovery Rate	0.0757	0.1155	0.1275	0.1116	0.4104
False Negative Rate	0.1972	0.1623	0.1310	0.1489	0.4219
Accuracy	0.8530	0.8607	0.8743	0.8704	0.5919
F1 Score	0.8593	0.8605	0.8708	0.8694	0.5838

**Table 30:** Ensemble model GALGO feature selection in Siglo XXI Male dataset: confusion matrix blind test measure values.

Measure	KNN	Nearcent	LR	SVM	NN
Sensitivity	0.8373	0.6856	0.6859	0.8235	0.8443
Specificity	0.8778	0.7258	0.7077	0.8837	0.8989
Precision	0.9267	0.8867	0.8733	0.9333	0.9400
Negative Predictive Value	0.7453	0.4245	0.4340	0.7170	0.7547
False Positive Rate	0.1222	0.2742	0.2923	0.1163	0.1011
False Discovery Rate	0.0733	0.1133	0.1267	0.0667	0.0600
False Negative Rate	0.1627	0.3144	0.3141	0.1765	0.1557
Accuracy	0.8516	0.6953	0.6914	0.8438	0.8633
F1 Score	0.8797	0.7733	0.7683	0.8750	0.8896

**Table 31:** Ensemble model GALGO feature selection in Siglo XXI Female dataset: confusion matrix blind test measure values.

Measure	KNN	Nearcent	LR	SVM	NN
Sensitivity	0.7658	0.6804	0.7719	0.7000	0.7748
Specificity	0.8926	0.7853	0.9110	0.8786	0.8993
Precision	0.8416	0.6535	0.8713	0.8317	0.8515
Negative Predictive Value	0.8365	0.8050	0.8365	0.7736	0.8428
False Positive Rate	0.1074	0.2147	0.0890	0.1214	0.1007
False Discovery Rate	0.1584	0.3465	0.1287	0.1683	0.1485
False Negative Rate	0.2342	0.3196	0.2281	0.3000	0.2252
Accuracy	0.8385	0.7462	0.8500	0.7962	0.8462
F1 Score	0.8019	0.6667	0.8186	0.7602	0.8113

**Table 32:** Ensemble model LASSO feature selection in Siglo XXI Overall/Male/Female datasets: confusion matrix blind test measure values.

Measure	Overall	Male	Female
Sensitivity	0.8760	0.8443	0.7500
Specificity	0.8801	0.8989	0.8851
Precision	0.8725	0.9400	0.8317
Negative Predictive Value	0.8835	0.7547	0.8239
False Positive Rate	0.1199	0.1011	0.1149
False Discovery Rate	0.1275	0.0600	0.1683
False Negative Rate	0.1240	0.1557	0.2500
Accuracy	0.8781	0.8633	0.8269
F1 Score	0.8743	0.8896	0.7887

**Table 33:** Summary ranges for the datasets.

Dataset	Controls—Cases	Table
Overall	Controls and Cases	Table 34
Male	Controls and Cases	Table 35
Female	Controls and Cases	Table 36
Overall	Controls	Table 37
Male	Controls	Table 38
Female	Controls	Table 39
Overall	Cases	Table 40
Male	Cases	Table 41
Female	Cases	Table 42

**Table 34:** Ranges for the Overall dataset (Controls and Cases).

Feature	Min	Lower Quartile	Median	Upper Quartile	Max
Age	30	45	52	60	82
WHR	0.72	0.87	0.92	0.97	1.11
BMI	17.00	25.26	27.90	31.24	40.20
Urea	6	24	28	36	54
Creatinine	0.36	0.68	0.79	0.93	1.30
Cholesterol	75.00	167.00	195.05	230.00	324.10
HDL	10	35	43	52	77
LDL	45.0	112.0	136.0	162.9	239.0
Triglycerides	32	116	156	219	371
TCHOLU	82	161	187	215	296
HDLU	12	36	44	52	76
LDLU	45	107	127	149	212
TGU	32	110	148	207	350
SBP	80	110	120	130	160
SBPU	80	110	120	130	160
DBP	50	70	80	85	105
DBPU	55	70	80	80	95

**Table 35:** Ranges for the Male dataset (Controls and Cases).

Feature	Min	Lower Quartile	Median	Upper Quartile	Max
Age	30	44	50	59	81
WHR	0.82	0.92	0.95	0.99	1.09
BMI	18.400	25.245	27.400	29.950	36.810
Urea	6	24	30	36	54
Creatinine	0.44	0.77	0.88	1.01	1.37
Cholesterol	75.00	160.50	186.00	219.05	303.10
HDL	10.1	32.0	40.0	47.0	69.1
LDL	45.0	109.0	131.0	157.0	225.9
Triglycerides	43.00	115.20	155.00	221.55	380.00
TCHOLU	82	155	180	207	285
HDLU	15	33	40	48	70
LDLU	45	102	125	145	203
TGU	43	112	150	210	357
SBP	90.0	110.5	120.0	130.0	158.0
SBPU	80	110	120	130	160
DBP	50	69	76	85	105
DBPU	55	69	76	80	95

**Table 36:** Ranges for the Female dataset (Controls and Cases).

Feature	Min	Lower Quartile	Median	Upper Quartile	Max
Age	30	47	54	60	79
WHR	0.71	0.84	0.89	0.93	1.06
BMI	17.20	25.28	28.60	32.46	43.21
Urea	9	24	28	34	49
Creatinine	0.360	0.625	0.720	0.810	1.080
Cholesterol	100.0	177.0	205.0	237.0	325.1
HDL	12.00	38.05	46.70	56.00	82.00
LDL	60.0	116.0	140.0	167.9	243.9
Triglycerides	32.00	116.00	156.00	216.05	364.00
TCHOLU	96	168	193	220	296
HDLU	16	40	48	57	82
LDLU	49	109	130	153	219
TGU	32	110	144	204	341
SBP	80	110	120	130	160
SBPU	80	110	120	130	160
DBP	50	70	80	85	105
DBPU	50	70	80	85	100

**Table 37:** Ranges for the Overall dataset (Controls only).

Feature	Min	Lower Quartile	Median	Upper Quartile	Max
Age	34	43	47	53	68
WHR	0.74	0.87	0.92	0.96	1.09
BMI	18.00	24.90	27.10	29.65	36.40
Urea	12	24	28	32	43
Creatinine	0.42	0.70	0.79	0.91	1.22
Cholesterol	87.0	162.0	186.0	214.5	291.0
HDL	17	38	45	53	75
LDL	45	108	128	151	214
Triglycerides	32	101	134	181	299
TCHOLU	87.0	162.0	186.0	214.5	291.0
HDLU	17	38	45	53	75
LDLU	45	108	128	151	214
TGU	32	101	134	181	299
SBP	90	110	119	127	152
SBPU	90	110	119	127	152
DBP	46	66	70	80	100
DBPU	46	66	70	80	100

**Table 38:** Ranges for the Male dataset (Controls only).

Feature	Min	Lower Quartile	Median	Upper Quartile	Max
Age	34	42	46	51	64
WHR	0.83	0.91	0.94	0.97	1.05
BMI	19.10	25.25	27.30	29.45	35.60
Urea	13	24	28	32	43
Creatinine	0.440	0.750	0.855	0.965	1.270
Cholesterol	87	159	181	208	281
HDL	15	34	41	49	71
LDL	45.0	105.0	126.0	146.5	203.0
Triglycerides	44	107	140	192	318
TCHOLU	87	159	181	208	281
HDLU	15	34	41	49	71
LDLU	45.0	105.0	126.0	146.5	203.0
TGU	44	107	140	192	318
SBP	90	111	120	128	152
SBPU	90	111	120	128	152
DBP	55	65	70	78	95
DBPU	55	65	70	78	95

**Table 39:** Ranges for the Female dataset (Controls only).

Feature	Min	Lower Quartile	Median	Upper Quartile	Max
Age	34.0	43.0	49.0	54.5	71.0
WHR	0.65	0.81	0.87	0.93	1.11
BMI	17.20	24.50	26.80	29.95	38.10
Urea	11	21	26	32	47
Creatinine	0.42	0.62	0.72	0.80	1.06
Cholesterol	100	167	195	220	291
HDL	19.0	43.0	51.0	59.5	84.0
LDL	60	111	132	155	221
Triglycerides	32	96	125	158	244
TCHOLU	100	167	195	220	291
HDLU	19.0	43.0	51.0	59.5	84.0
LDLU	60	111	132	155	221
TGU	32	96	125	158	244
SBP	90	109	113	124	145
SBPU	90	109	113	124	145
DBP	50	68	71	80	95
DBPU	50	68	71	80	95

**Table 40:** Ranges for the Overall dataset (Cases only).

Feature	Min	Lower Quartile	Median	Upper Quartile	Max
Age	31	50	57	63	82
WHR	0.75	0.88	0.92	0.97	1.10
BMI	17.48	25.81	28.95	32.68	42.96
Urea	9	26	30	39	58
Creatinine	0.26	0.67	0.79	0.95	1.35
Cholesterol	91.00	176.00	207.10	242.05	340.00
HDL	10	33	41	50	75
LDL	51.0	118.0	143.0	175.0	256.9
Triglycerides	41.00	139.00	185.00	253.05	417.10
TCHOLU	91	161	187	215	296
HDLU	12.0	35.5	42.0	52.0	76.0
LDLU	49	106	127	148	209
TGU	41	123	164	230	387
SBP	70	110	130	140	180
SBPU	80	110	120	130	160
DBP	65	80	85	90	105
DBPU	70	80	80	90	100

**Table 41:** Ranges for the Male dataset (Cases only).

Feature	Min	Lower Quartile	Median	Upper Quartile	Max
Age	30	50	58	65	86
WHR	0.84	0.94	0.97	1.01	1.11
BMI	17.480	25.245	27.810	31.015	39.340
Urea	6	26	32	43	66
Creatinine	0.54	0.79	0.93	1.08	1.51
Cholesterol	91.00	165.05	194.10	237.10	340.00
HDL	10.10	30.10	36.70	44.05	63.00
LDL	51.0	113.0	138.0	168.5	250.9
Triglycerides	43.0	134.2	182.0	257.7	433.0
TCHOLU	91.0	151.0	180.0	204.5	284.0
HDLU	16	31	39	45	66
LDLU	51.0	99.0	122.0	143.5	201.0
TGU	43.0	122.0	166.0	230.5	377.0
SBP	80	110	130	140	180
SBPU	80	110	120	130	160
DBP	65	80	85	90	105
DBPU	70	80	80	90	100

**Table 42:** Ranges for the Female dataset (Cases only).

Feature	Min	Lower Quartile	Median	Upper Quartile	Max
Age	34.0	50.5	56.0	63.0	79.0
WHR	0.73	0.85	0.89	0.93	1.05
BMI	17.84	26.53	29.77	33.61	43.96
Urea	6	24	30	36	54
Creatinine	0.36	0.63	0.72	0.82	1.10
Cholesterol	108.00	184.05	213.55	245.60	333.10
HDL	12.0	36.0	44.1	53.0	78.0
LDL	62.00	123.00	147.95	179.90	264.90
Triglycerides	41.00	141.05	188.25	248.90	407.00
TCHOLU	96.0	168.5	192.0	220.0	296.0
HDLU	16	38	46	54	78
LDLU	49.0	108.0	129.5	150.0	209.0
TGU	41.0	123.0	164.0	229.5	387.0
SBP	90	115	130	140	175
SBPU	80	110	120	130	160
DBP	65	80	85	90	105
DBPU	70	80	80	90	100

**Table 43:** Related works.

Title	Technique	Measures
Use of Non-invasive Parameters and Machine Learning Algorithms for Predicting Future Risk of T2DM: A Retrospective Cohort Study of Health Data From Kuwait [66]	KNN, LR and SVM	AUC KNN (0.83), AUC LR (0.74), AUC SVM (0.73)
The potential for a two-stage diabetes risk algorithm combining non-laboratory-based scores with subsequent routine non-fasting blood tests: results from prospective studies in older men and women [67]	LR	AUC LR (0.77)
Sex Differences in Plasma MicroRNA Biomarkers of Early and Complicated Diabetes Mellitus in Israeli Arab and Jewish Patients [68]	Sequence Detection System (SDS) 2.3 (Applied Biosystems), Microsoft Excel, WinSTAT, StatPlus Mac LE (AnalystSoft, Walnut, CA, USA) software, and Student’s *t*-test.	Accuracy (0.77) and Sensitivity (0.79)
Biochemical investigation of sex-specific association between insulin resistance and inflammatory biomarkers in types 2 diabetic patients [69]	Multiple linear regression, ANOVA	BMI in relation to insulin resistance: Pearson’s correlation coefficient 0.9188 in Males and 0.9694 in Females; coefficient of determination 0.8442 in Males and 0.9398 in Females; confidence interval 0.8349 to 0.9610 in Males and 0.9361 to 0.9855 in Females, for a *p*-value lower than 0.0001 in all cases
This work	Feature selection: RFE with AIC, LASSO, and GA with GALGO. ML models: LR, KNN, Nearcent, ANN, and SVM	Table 26, Table 27, Table 28, Table 29, Table 30, Table 31 and Table 32

## Data Availability

No new data were created or analyzed in this study. Data sharing is not applicable to this article.

## Abbreviations

The following abbreviations are used in this manuscript:

ACARBO_MG_DIA	Acarbose prescribed in milligrams
ACARBOSA	If patient has Acarbose treatment
Age	Age of the patient in years
Age DX (cases)	Years with diabetes disease diagnosed
AIC	Akaike Information Criterion
ANN	Artificial Neural Networks
AUC	Area Under the Curve
BA	Biological Age
BIC	Bayesian Information Criterion
BMI	Body Mass Index
CA	Chronological Age
CHOL	Fat-like substance that is found in all cells of the patient body
CIC	Corrected Information Criterion
CK-MB	Creatine Kinase-MB
Creatinine	Waste product produced by muscles as part of regular daily activity
DBP	Diastolic Blood Pressure
DBPU	Diastolic Blood Pressure (uncorrected by medication)
DIC	Deviance Information Criterion
DKD	Diabetic Kidney Disease
DR	Diabetic Retinopathy
DT	Decision Tree
Edu	Education Level
FFANN	Feed-Forward Artificial Neural Network
FIC	Focused Information Criterion
FNN	Feed-Forward Neural Network
FPG	Fasting Plasma Glucose
FS	Forward Selection
GA	Genetic Algorithms
GFR	Glomerular Filtration Rate
GLIBEN_MG_DIA	Glibenclamide prescribed in milligrams
GLIBENCLAMIDA	If patient has Glibenclamide treatment
GLU (mg/dL)	Glucose levels
GNs	Glomerulopathies
HA-TX	Subject under Hypertension Treatment
HbA1c	Glycosylated Hemoglobin
HDL	High Density Lipoprotein
HDLU	High Density Lipoprotein (uncorrected by medication)
id	Consecutive identification number in dataset
IgAN	Immunoglobulin A Nephropathy
INSUL_UI_DIA	Insulin prescribed in milligrams
INSULINA	If patient has Insulin treatment
IR	Insulin Resistance
KAI	Kidney Age Index
KNN	K-Nearest Neighbors
LASSO	Least Absolute Shrinkage and Selection Operator
LDL	Low Density Lipoprotein
LDLU	Low Density Lipoprotein (uncorrected by medication)
Lipids treatment	Lipid levels in treatment
LIPIDS-TX	Subject under Lipids Treatment
LR	Logistic Regression
METFOR_MG_DIA	Metformin prescribed in milligrams
METFORMINA	If patient has Metformin treatment
ML	Machine Learning
MS	Mass Spectrometry
NA	Null or not available data
Nearcent	Nearest Centroid
NLP	Natural Language Processing
OPN	Osteopontin
PIOGLI_MG_DIA	Pioglitazone prescribed in milligrams
PIOGLITAZONA	If patient has Pioglitazone treatment
plate_info	Hospital patient id
RF	Random Forest
RFE	Recursive Feature Elimination
ROSIGLI_MG_DIA	Rosiglitazone prescribed in milligrams
ROSIGLITAZONA	If patient has Rosiglitazone treatment
Sal	Salary
SBP	Systolic Blood Pressure
SBPU	Systolic Blood Pressure (uncorrected by medication)
Sex	Patients sex
SVM	Support Vector Machines
T2DM	Type 2 Diabetes Mellitus
TCHOLU	Total Cholesterol (uncorrected by medication)
TGU	Triglycerides (uncorrected by medication)
TIPO_COMPLICACION	T2DM complications
DE DT2	
Urea	Waste product resulting from the breakdown of protein in the patient body
WHR	Waist-to-Hip Ratio
